# Old wild wolves: ancient DNA survey unveils population dynamics in Late Pleistocene and Holocene Italian remains

**DOI:** 10.7717/peerj.6424

**Published:** 2019-03-27

**Authors:** Marta Maria Ciucani, Davide Palumbo, Marco Galaverni, Patrizia Serventi, Elena Fabbri, Gloria Ravegnini, Sabrina Angelini, Elena Maini, Davide Persico, Romolo Caniglia, Elisabetta Cilli

**Affiliations:** 1 Laboratories of Physical Anthropology and Ancient DNA, Department of Cultural Heritage, University of Bologna, Ravenna, Italy; 2 Natural History Museum of Denmark, Copenhagen, Denmark; 3 Ente di Gestione per i Parchi e la Biodiversità Emilia Orientale, Monteveglio, Italy; 4 Conservation Unit, WWF Italia, Rome, Italy; 5 Unit for Conservation Genetics (BIO-CGE), Italian Institute for Environmental Protection and Research (ISPRA), Ozzano dell’Emilia, Bologna, Italy; 6 Department of Biological, Geological & Environmental Sciences—BiGeA, University of Bologna, Bologna, Italy; 7 Department of Pharmacy and Biotechnology, University of Bologna, Bologna, Italy; 8 ArcheoLaBio—Research Centre for Bioarchaeology, Department of History and Culture, University of Bologna, Ravenna, Italy; 9 Department of Chemistry, Life Sciences and Environmental Sustainability, University of Parma, Parma, Italy

**Keywords:** mtDNA, Ancient DNA, HVR1 variability, *Canis lupus*, Wolf, Italian wolf, Control region, Population genetics, Canid

## Abstract

**Background:**

The contemporary Italian wolf (*Canis lupus italicus*) represents a case of morphological and genetic uniqueness. Today, Italian wolves are also the only documented population to fall exclusively within the mitochondrial haplogroup 2, which was the most diffused across Eurasian and North American wolves during the Late Pleistocene. However, the dynamics leading to such distinctiveness are still debated.

**Methods:**

In order to shed light on the ancient genetic variability of this wolf population and on the origin of its current diversity, we collected 19 Late Pleistocene-Holocene samples from northern Italy, which we analyzed at a short portion of the hypervariable region 1 of the mitochondrial DNA, highly informative for wolf and dog phylogenetic analyses.

**Results:**

Four out of the six detected haplotypes matched the ones found in ancient wolves from northern Europe and Beringia, or in modern European and Chinese wolves, and appeared closely related to the two haplotypes currently found in Italian wolves. The haplotype of two Late Pleistocene samples matched with primitive and contemporary dog sequences from the canine mitochondrial clade A. All these haplotypes belonged to haplogroup 2. The only exception was a Holocene sample dated 3,250 years ago, affiliated to haplogroup 1.

**Discussion:**

In this study we describe the genetic variability of the most ancient wolf specimens from Italy analyzed so far, providing a preliminary overview of the genetic make-up of the population that inhabited this area from the last glacial maximum to the Middle Age period. Our results endorsed that the genetic diversity carried by the Pleistocene wolves here analyzed showed a strong continuity with other northern Eurasian wolf specimens from the same chronological period. Contrarily, the Holocene samples showed a greater similarity only with modern sequences from Europe and Asia, and the occurrence of an haplogroup 1 haplotype allowed to date back previous finding about its presence in this area. Moreover, the unexpected discovery of a 24,700-year-old sample carrying a haplotype that, from the fragment here obtained, falls within the canine clade A, could represent the oldest evidence in Europe of such dog-rich clade. All these findings suggest complex population dynamics that deserve to be further investigated based on mitochondrial or whole genome sequencing.

## Introduction

The gray wolf (*Canis lupus*) is the most widespread large carnivore of the Holarctic region (Mech & Boitani, 2010). Its high mobility and dispersal ability allow it to cover 100 of kilometers (Fritts, 1983; Valière et al., 2003; Ciucci et al., 2009; Andersen et al., 2015) and favor gene flow between populations. Nonetheless, the existence of differentiated contiguous wolf populations linked to habitat and prey specializations has been well documented (Carmichael et al., 2001; Geffen, Anderson & Wayne, 2004; Musiani et al., 2007). In addition, in the last two centuries this species experienced complex and dramatic demographic changes (Breitenmoser, 1998), and only during the last few decades has it successfully recovered and expanded thanks to legal protection and socio-ecological changes (Chapron et al., 2014). This combination of factors makes the wolf phylogeographic history complex and difficult to disentangle from contemporary genetic patterns (Randi, 2011; Ersmark et al., 2016). Furthermore, in Eurasia, the wide spatio-temporal gaps between the ancient samples so far analyzed has provided us with a picture of wolf population dynamics in the last 50,000 years, but given the complexity of population migration and admixture, this was most likely more complex and deserves to be analyzed with additional sampling and genotyping efforts on ancient remains (Pilot et al., 2010; Ersmark et al., 2016).

To date, a number of studies have tried to investigate the species history analyzing mitochondrial DNA (mtDNA; Pilot et al., 2010; Thalmann et al., 2013; Ersmark et al., 2016; Koblmuller et al., 2016) and nuclear genomes (vonHoldt et al., 2011; Skoglund et al., 2015; Fan et al., 2016) of ancient and modern specimens. In particular, the study by Pilot et al. (2010) on the mtDNA hypervariable region (HVR) suggested the existence of two main and distinct wolf mitochondrial haplogroups (known as Hg1 and Hg2) in the Eurasian wolf populations.

Whereas Pleistocene specimens from Beringia—that based on extensive morphological records were described as a hypercarnivorous wolf ecomorph—carried only Hg2 haplotypes (Leonard et al., 2007), in Europe, the once dominant wolf haplogroup (Hg2)—observed since 40,000 years ago—was largely replaced by Hg1 in the Holocene. This resulted in the presence of both haplogroups at variable frequencies in modern populations, but with Hg1 haplotypes reaching an average frequency of 76% (Pilot et al., 2010). However, an exception to this pattern is represented by the Italian wolf population (*Canis lupus italicus*, Giuseppe Altobello, 1921; Montana et al., 2017a), all belonging exclusively to Hg2 (Pilot et al., 2010).

In particular, the Italian wolves currently show clear morphological (Nowak & Federoff, 2002) and genetic uniqueness worldwide. More than 1,000 Italian wolf specimens studied (Italian Institute for Environmental Protection and Research -ISPRA- database) during the last 20 years showed to be characterized by the presence of only two distinct mtDNA haplotypes (Randi et al., 2000; Boggiano et al., 2013; Montana et al., 2017a). Additional studies showed that this population is phylogenetically close to Late Pleistocene wolves (based both on 582 base pair (bp) of the d-loop region, Ersmark et al., 2016; and on the full mtDNA, Thalmann et al., 2013), and exhibits distinct nuclear allele frequencies (Pilot et al., 2010; vonHoldt et al., 2011; Montana et al., 2017a).

Initial studies on modern Italian wolves hypothesized that their distinctiveness could be attributed to the recent genetic isolation and drift linked to the extreme human-driven bottleneck occurred in the mid-1900s (Cagnolaro et al., 1974; Randi, Lucchini & Francisci, 1993), which resulted in only 100–200 individuals surviving in the Central and Southern Apennines (Zimen & Boitani, 1975; Mech & Boitani, 2010). Conversely following works suggested that their demographic decline could have rather begun at the end of the last glacial maximum (LGM), with a progressive reduction through time similar to other Eurasian populations (Fan et al., 2016), and only exacerbated by the more extreme historical bottleneck of the Italian population (Lucchini, Galov & Randi, 2004; vonHoldt et al., 2011; Boggiano et al., 2013; Pilot et al., 2014; Montana et al., 2017a, 2017b).

Despite a fair number of studies on modern Italian wolves, only a single study to date has tried to investigate the genetic variability of ancient *Canis lupus* from this Peninsula (Verginelli et al., 2005). Nevertheless, their analyses of a few samples dated from 15,000 to 3,000 years ago showed, for most of them, an uncertain attribution to dogs or wolves, either morphologically, genetically or both.

Therefore, in this study we analyzed 19 ancient canid samples collected in the northern Apennines and in the Po Valley (Italy), dated between 25,000 and 890 years ago, that we sequenced at the HVR1 of the mitochondrial DNA, aiming to: (1) investigate the genetic variability of the ancient Italian wolf population; and (2) trace the origins of the current genetic uniqueness of Italian wolves.

## Materials and Methods

### Sample collection and dating

The skeletal remains (bones and teeth) of 19 Italian canids from Late Pleistocene, Bronze Age and the Middle Ages (Table 1) were collected from three different sites located in northern Italy (See Fig. S2 in Supplemental Article S1 for the map of the archaeological sites). Two of them, the Cava Filo site (San Lazzaro di Savena, Bologna) and the Monterenzio Vecchio site (Monterenzio, Bologna), are situated in the northern Apennines, while the third site is on the alluvial bar of the Po River (Province of Cremona). Samples were provided, respectively, by the Prehistoric Museum “Luigi Donini” (San Lazzaro di Savena, Bologna), by the Archaeological Museum “Luigi Fantini” (Monterenzio, Bologna) and by the University of Parma. From the collections gathered from the Cava Filo site, we selected 16 samples, ascribable to a chronological range comprised between 25,000 and 17,220 years ago (Table 1). We also selected two samples from the archaeological site of Monterenzio Vecchio dated at the beginning of the Late Bronze Age, around 3,250 years ago (Guerra et al., 2010; Maini, 2012). The last sample analyzed was a wolf skull emerged from an alluvial bar of the Po River and radiocarbon dated to 890 years ago. Two of our samples, OWW9 and OWW19, were directly radiocarbon dated, whereas all the others were dated based on the stratigraphy and material culture of the archaeological site they were found, or on the availability of C14 dating of other samples in the same stratigraphic unit or in close proximity to them (See Supplemental Article S1, Table 1 and Table S1 for information about specimens, dates and sampling sites).

**Table 1:** List of the specimens analyzed in this study with pertinent information.

Museum ID	Sample ID	Site	Museum	Sample type	Age (BP)	Dating Reference
124	OWW1	Cava Filo	Prehistoric Museum “Luigi Donini”	tooth (molar)	22,285–17,869	Pasini (1970), Paronuzzi et al. (2018)
547	OWW2	Cava Filo	Prehistoric Museum “Luigi Donini”	tooth (molar)	22,285–17,869	Pasini (1970), Paronuzzi et al. (2018)
556	OWW3	Cava Filo	Prehistoric Museum “Luigi Donini”	tooth (canine)	22,285–17,869	Pasini (1970), Paronuzzi et al. (2018)
557	OWW4	Cava Filo	Prehistoric Museum “Luigi Donini”	radius (distal part)	22,285–17,869	Pasini (1970), Paronuzzi et al. (2018)
06–027	OWW5	Cava Filo	Prehistoric Museum “Luigi Donini”	ulna	23,940	Paronuzzi et al. (2018)
07–201	OWW6	Cava Filo	Prehistoric Museum “Luigi Donini”	metatarsal	17,550	Paronuzzi et al. (2018)
08–057	OWW7	Cava Filo	Prehistoric Museum “Luigi Donini”	front tooth	17,550	Paronuzzi et al. (2018)
09–049	OWW8	Cava Filo	Prehistoric Museum “Luigi Donini”	metapodium	23,940	Paronuzzi et al. (2018)
09–050	OWW9	Cava Filo	Prehistoric Museum “Luigi Donini”	radius	24,700	This study
09–072	OWW10	Cava Filo	Prehistoric Museum “Luigi Donini”	phalanx	23,940	Paronuzzi et al. (2018)
11–018	OWW11	Cava Filo	Prehistoric Museum “Luigi Donini”	metapodium	23,940	Paronuzzi et al. (2018)
11–035	OWW12	Cava Filo	Prehistoric Museum “Luigi Donini”	metapodium	17,550	Paronuzzi et al. (2018)
11–055	OWW13	Cava Filo	Prehistoric Museum “Luigi Donini”	metapodium	23,940	Paronuzzi et al. (2018)
11–083	OWW14	Cava Filo	Prehistoric Museum “Luigi Donini”	humerus (distal part)	23,940	Paronuzzi et al. (2018)
11–089	OWW15	Cava Filo	Prehistoric Museum “Luigi Donini”	metapodium (distal part)	23,940	Paronuzzi et al. (2018)
11–108	OWW16	Cava Filo	Prehistoric Museum “Luigi Donini”	metapodium	17,550	Paronuzzi et al. (2018)
MV 07	OWW17	Monterenzio Vecchio	Archaeological Museum “Luigi Fantini”	tooth (molar)	3,250	Guerra et al. (2010)
MV 2005	OWW18	Monterenzio Vecchio	Archaeological Museum “Luigi Fantini”	metatarsal	3,250	Guerra et al. (2010)
MSDP 348	OWW19	Po River	Univesity of Parma	skull	890	This study

**Note:**

For each sample the IDs are indicated together with the archaeological site, museums, specimen type, age and dating reference. To facilitate the temporal placement of the samples in the text and in the analyses, the age here indicated represents the average of the data range provided from C14 datation (for Cava Filo and Po River) or from stratigraphy and material culture (for Monterenzio Vecchio) (see Table S1 for detailed information about radiocarbon analyses and age estimation).

### Ancient DNA standards and DNA extraction

All laboratory procedures followed strict and appropriate criteria, selected among those suggested by Cooper & Poinar (2000), to support the authenticity of the results and to prevent contamination by exogenous DNA. The decontamination, drilling, DNA extraction and pre-PCR set up of the ancient samples were performed in physically separated and designated areas (pre-PCR lab) at the Laboratories of Physical Anthropology and Ancient DNA of the Department of Cultural Heritage (University of Bologna, Ravenna Campus) with high standards of sterility and exclusively reserved for ancient DNA analysis (Fulton, 2012; Knapp et al., 2012). The pre-PCR lab is organized in separate rooms, dedicated to the different phases of the workflow, where modern samples have never been introduced. All the surfaces of non-disposable equipment and instruments were cleaned by bleach and ethanol or solely by DNA-ExitusPlus™ (Applichem Inc., Omaha, NE, USA). All the reagents used during the DNA extraction or PCR set-up, as well as all the plastic labware, were exposed to UV radiation for 60 min prior to their use (except for DNA polymerase, primers and dNTPs). Suitable disposable clothing (full body suit, hair cap, boots, face mask, face shield, arm covers and two pairs of gloves) were worn during the analyses of ancient samples in the pre-PCR facility. Moreover, prior to DNA isolation, all samples were superficially decontaminated by slight abrasion with a sterile diamond-drill to remove the superficial layers of the sample (one to two mm), then exposed to UV radiation on each side for 60 min.

The DNA was isolated using a silica-based protocol (Serventi et al., 2018), slightly modified from Dabney, Meyer & Paabo (2013) and Allentoft et al. (2015) (See Supplemental Article S1 for details about extraction protocol). In order to avoid the risk of exogenous DNA and cross-contaminations, the samples were processed in small batches of five to six samples. At the end of the extraction DNA was quantified using a Qubit® dsDNA HS (High Sensitivity) Assay Kit (Invitrogen™Life Technologies, Carlsbad, CA, USA). All the samples were extracted and amplified at least twice in order to verify the authenticity of the results. To avoid contaminations by amplicons, PCR runs and downstream analyses were conducted in a physically separated facility, dedicated to post-PCR procedures. In addition, during each day the personnel were only allowed to move from the pre-PCR lab to the post-PCR lab.

### DNA amplification and sequencing

Given the expected highly fragmented state of the endogenous DNA preserved in the samples (Hofreiter et al., 2001), we decided to amplify a 57 bp fragment (99 bp with primers) of the HVR1 region included between nucleotide positions 15,615–15,671 of the Italian wolf mitochondrial genome (Genbank accession number KU644662) using primers from Stiller et al. (2006). The amplicon sequences, despite their limited size, include the majority of the informative nucleotide positions (30 polymorphic sites) of the mtDNA control region of dogs and wolves, already tested for phylogenetic purposes in several studies concerning ancient canids (Stiller et al., 2006; Germonpré et al., 2009; Pilot et al., 2010; Ersmark et al., 2016). Samples were also tested for a longer region of 361 bp (404 bp with primers) spanning bases 15,431–15,792, by means of the amplification of three overlapping fragments (Leonard, Vilà & Wayne, 2005; Ersmark et al., 2016).

Amplicons were checked in agarose gel, then purified and sequenced (See Supplemental Article S1 for details about PCR reactions and sequencing). Due to the intrinsic characteristic of ancient sequences to be highly damaged, nucleotide substitutions can occur mainly as deaminations, that determine a transition from C to T and G to A (Hansen et al., 2001), which mainly occurs at the end of the molecule (Briggs et al., 2007). For this reason, multiple extractions, independent amplifications and further sequencing were performed in order to improve the detection of the damaged sites, comparing at least two electropherograms of each sample.

Sequences are available on GenBank (accession numbers: MH085470–MH085479 & MH593822).

### Haplotypes identification and phylogenetic analyses

All sequences obtained from the extracted samples were visualized, edited and aligned in Unipro UGENE 1.27 (Okonechnikov, Golosova & Fursov, 2012). A database of mtDNA control region sequences including the major modern wolf populations and dog breeds was obtained from Montana et al. (2017b), gathering 127 sequences (Table S2), and was used to compare the haplotypes obtained in this study to the current genetic variability of wolves and dogs. Samples of uncertain geographical origin or species attribution were excluded from the database. The nomenclature of the control region used from now on is based on that proposed by Montana et al. (2017b) referring to the control region (See Table S2 for the codes and the area of sampling). An additional database of 108 ancient dog and wolf sequences from Europe, Asia, America and Oceania, dated from 49,000 to 800 years ago was created (Table S3). All the downloaded sequences were trimmed down to match the shorter amplified fragment (57 bp) and, when the length of the sequences allowed it, to a stretch of 330 bp, in order to compare the longer fragment of 361 bp here obtained with the data available in literature. We then created four reference alignments: the first one (Alignment A, 57 bp) consisted of sequences from the main extant Eurasian wolf populations, the second (Alignment B, 57 bp) with ancient sequences and the two current Italian wolf haplotypes, and the third one (Alignment C, 330 bp) with the longer modern and ancient sequences. A fourth alignment (Alignment D, 239 bp) was built to include in the analysis the five ancient Italian samples from the previous study by Verginelli et al. (2005), whose attribution as dogs or wolves was uncertain. Due to the size of Italian sequences this alignment was trimmed to 239 bp (Table S2 and Table S3).

The software DnaSP v.5.10.01 (Librado & Rozas, 2009) was used to identify identical sequences and to collapse them into unique haplotypes. Median-Joining (MJ) networks were created by the software PopART (Leigh & Bryant, 2015) using Alignment A and Alignment B (*ε* = 0).

A maximum likelihood (ML) phylogenetic tree was reconstructed with the software MEGA7 (Kumar, Stecher & Tamura, 2016) using the Alignment C, by setting the substitution model HKY+I+G, assessed by JModeltest2 (Darriba et al., 2012), running 1,000 bootstrap repetitions and using a coyote sequence as an outgroup (*Canis latrans,* GenBank acc. number: DQ480509). MrBayes v. 3.2 (Huelsenbeck & Ronquist, 2001) was used to generate a Bayesian tree using the Alignment D and the software run for 10^7^ generations, with a 10% burn-in and a sampling frequency each 1,000 iteration. Tracer v. 1.6 (Rambaut et al., 2018) was used to check the convergence of parameters result from the two runs and the final tree was visualized with FigTree v. 1.4.3 (Rambaut, 2012).

## Results

### Authenticity of the results and successful rate of ancient DNA analysis

The procedures performed and the strict criteria chosen for this study to estimate the reliability of aDNA results (see ‘Materials and Methods’) make us confident concerning the authenticity of the retrieved sequences presented below (Gilbert et al., 2005). Moreover, the validity was supported by the following evidences: (i) no contamination was observed in any of the blank extractions or negative PCR controls included in each reaction and (ii) all consensus haplotypes were determined by both forward and reverse sequences and also using multiple replicates (i.e., samples from independent extracts and amplifications performed at different times).

Regarding the 57 bp fragment, we successfully obtained the consensus sequences from 11 out of 19 samples that fully complied with the selected authentication criteria and thus were used for the downstream analyses. Considering the remaining eight samples, it was not possible to obtain a successful amplification for three of them, whereas the sequences of the other five samples were discarded due to discrepancies between the resulting electropherograms. Although this could be due to *post mortem* damage, we conservatively chose to exclude them to ensure the fidelity of the dataset. Furthermore, as a result of the high fragmentation of the DNA, for only one sample (OWW9, dated 24,700-years-old) we were able to obtain the amplification of all three fragments (148 bp, 205 bp and 211 bp) for the longer region of 361 bp (See Materials and Methods). Our results and the high failure rate in the amplification of longer fragments confirm and support the diagenesis of DNA, in accord with the age of the samples.

### Haplotype variability

The 11 reliable sequences obtained for the 57 bp fragment were collapsed into six different haplotypes, all of which have previously been reported in the literature (Table 2).

**Table 2:** Haplotypes and haplogroups assignment of the samples based on the short sequences (57 bp) obtained in this study and their matches with the sequences available in GenBank.

ID sample	Age (BP)	mtDNA Haplogroup	Match—ancient samples from GenBank	Match—modern samples from GenBank	Haplotype ID (modern samples)
OWW4; OWW9	24,700–17,869	2	*C. lupus—*Germany 2.000—Stiller et al. (2006) (DQ852651)	97 modern sequences *C. l. familiaris*	D5–D6
			*C. lupus*—Russia 8.500*—Zhilin et al. (2014) (LM993795)	hybrid *C. lupus—*Iran (KC540925)	
				*C. lupus—*China (KX898354)	
OWW8; OWW11; OWW13; OWW15; OWW16	23,940–17,550	2	*C. lupus*—Belgium 26.200—Stiller et al. (2006) (DQ852650)	/	/
OWW12	17,550	2	*C. lupus—*Alaska 20.800—Thalmann et al. (2013) (KF661090)	*C. l. familiaris—*Bali street dog (HQ287728)	D104
			*C. lupus—*Russia 33.500—Thalmann et al. (2013) (KF661092)		
			*C. lupus—*Alaska 17.300*—Leonard et al. (2007)		
			*C. lupus*—Alaska 15.800*—Leonard et al. (2007)		
			*C. lupus—*Czech Republic 47.700—Stiller et al. (2006) (DQ852635)		
OWW17	3,250	2	/	*C. lupus*—Hungary (KP665919)	W39–D103
				*C. l. familiaris*—China (KJ139080)	
OWW18	3,250	1	/	11 modern sequences *C. lupus*—Iberian Peninsula	W20–W21
OWW19	890	2	/	*C. l. lupus*—Greece (AF115700)	W15

**Note:**

Matches with modern wolf (W) and dog (D) haplotypes follow the nomenclature based on Montana et al. (2017b). For further information see Table S2. Uncalibrated radiocarbon dates are shown by an asterisk.

The haplotype of five samples (OWW8, OWW11, OWW13, OWW15, OWW16—belonging to two different S.U., dated from 23,940 to 17,550 years ago) matched that reported from a Pleistocene wolf found in Belgium dated to 26,200 years ago (Stiller et al., 2006), and have not to date been reported in any modern sample. The 24,700-year-old sample OWW9, together with OWW4, matched the haplotype found in a Holocene specimen from Germany (Stiller et al., 2006) dated 2,000 years ago, and in a 8,500 BP dog coprolite from Russia (Zhilin et al., 2014). The same sequence was also retrieved in 97 globally-distributed modern dogs, in a modern Iranian wolf × dog hybrid (Aghbolaghi et al., 2014) and in a modern *Canis lupus* from China (Ersmark et al., 2016). The longer fragment recovered from OWW9 confirmed the above-mentioned correlations with modern samples, but it was not possible to compare it with ancient specimens due to their shorter fragments available. Given the results obtained, the analyses on this sample were nonetheless aimed at deepening the phylogenetic relationship with dogs, which highlighted that this haplotype fall within the canine clade A (Thai, Chung & Tran, 2017). The 17,550-years-old sample OWW12 shared the haplotype of several ancient wolves from Alaska, Russia and Czech Republic spanning from 15,000 to 47,000 years old (Stiller et al., 2006; Leonard et al., 2007; Thalmann et al., 2013) and of a modern street dog from Bali (Irion et al., 2005) (see Table 2). The three Holocene samples from Monterenzio Vecchio and Po River showed no similarity with any ancient samples reported in the literature: OWW17 had the same haplotype of a modern Hungarian wolf (held in a zoo and with unknown origin; GenBank accession number: KP665919) and of a Chinese dog (GenBank accession number: KJ139080), while OWW18 exhibited the same haplotype of some extant Iberian wolves and OWW19 corresponded with the Greek wolf haplotype W15 (Montana et al., 2017b).

### Phylogenetic analyses

**Figure 1:**
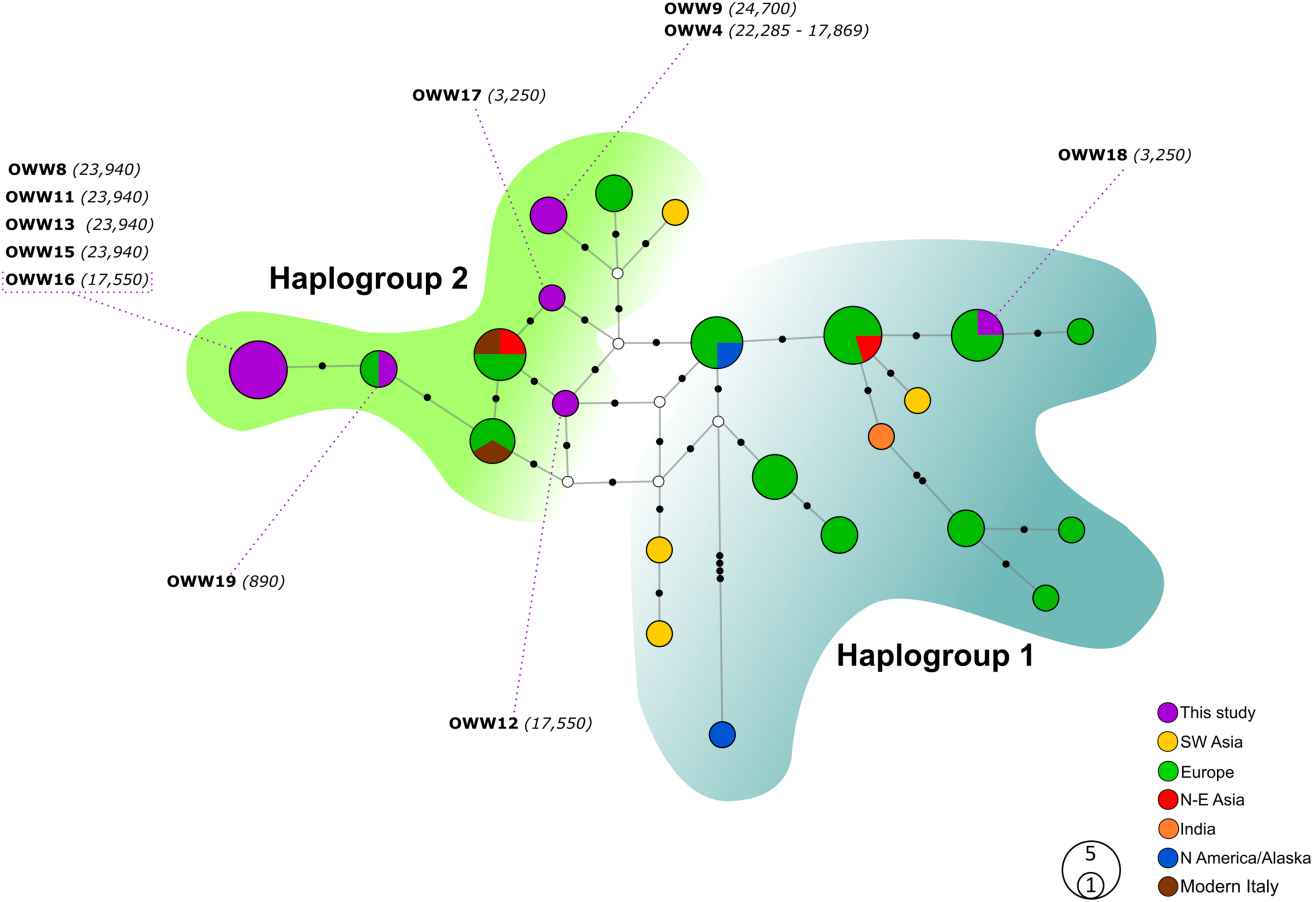
Median-Joining network based on Alignment A (57 bp sequences obtained in this study and modern Eurasian wolf populations). White circles represent median vectors; black circles correspond to nucleotide mutations. Haplotypes (circles) are colored according to their geographical provenience while the Italian (ancient and modern) haplotypes are represented by two different colors to discern them from the other European haplotypes. Hg2 is represented by the green area on the left, whereas Hg1 is encompassed by the shaded cyan area on the right. The date of each sample is reported in brackets and is intended in years before present (BP).

**Figure 2:**
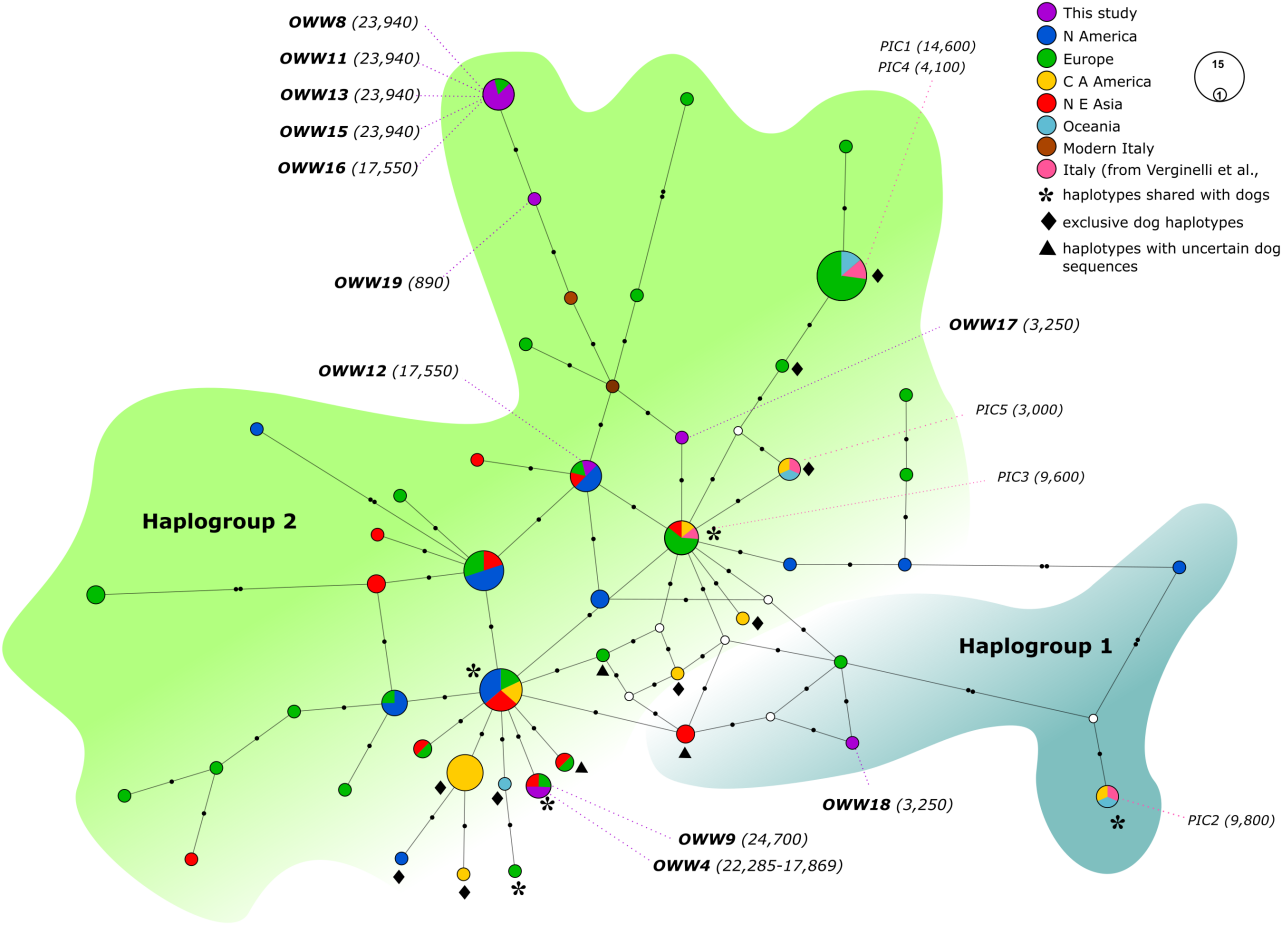
Median-Joining network based on Alignment B (57 bp) including ancient wolf and dog haplotypes plus the two extant Italian wolf haplotypes. When not specified the haplotypes depicted are referred to wolves. Diamonds highlight exclusive dog haplotypes; asterisks indicate the haplotypes shared between dogs and wolves and triangles designate the three haplotypes that present sequences with uncertain attribution to dogs or wolves. White circles represent median vectors, black circles correspond to nucleotide mutations. Italian samples analyzed by Verginelli et al. (2005) are also shown in the figure using the pink color. Haplotypes (circles) are colored according to their geographical provenience. Hg1 is represented by the cyan area on the right whereas Hg2 is represented by the green area on the top-left. The date of each sample is reported in brackets and is intended in years before present (BP).

The two MJ networks, despite being created from the 57 bp mtDNA alignments (A and B), confirmed the distinction between the two mitochondrial haplogroups Hg1 and Hg2 (Fig. 1 and Fig. 2, respectively) proposed on the longer fragment of the control region by Pilot et al. (2010). All our samples, with the only exception of the 3,250-year-old sample OWW18 belonging to Hg1, grouped within Hg2, but none of their haplotypes matched those found in modern Italian wolves. With the exception of OWW9 and OWW4, whose haplotype was closer to a Saudi Arabian and a North European modern wolf haplotype, all our Pleistocene sample haplotypes are placed only one mutational step far from the current Italian haplotypes (W14 and W16) and from the Greek haplotype W15, that we documented in Italy *c*. 890 years ago and is currently absent (Fig. 1). Nonetheless, most of the analyzed samples did not share haplotypes with any of the current wolf populations used for comparison (Fig. 1), reflecting that part of the variability observed in ancient samples has been lost through time.

When looking at the relationship with the other ancient samples available, the picture becomes richer, reconfirming the partition into two haplogroups but also the absence of any clear correspondence with geographic origins of samples (Fig. 2). As expected, most of our samples (eight out of 11) carried haplotypes shared with other ancient canid samples (Fig. 2). The three more recent samples OWW17, OWW18 and OWW19 were associated only with modern non-Italian haplotypes (Fig. 1) resulting in a mismatch with ancient samples in the network (Fig. 2). OWW17 and OWW19 (3,250 and 890 years old, respectively) belonged to Hg2 whereas OWW18 (3,250 years old) was the only one belonging to Hg1 and corresponding to modern Iberian haplotypes (W20–W21).

**Figure 3:**
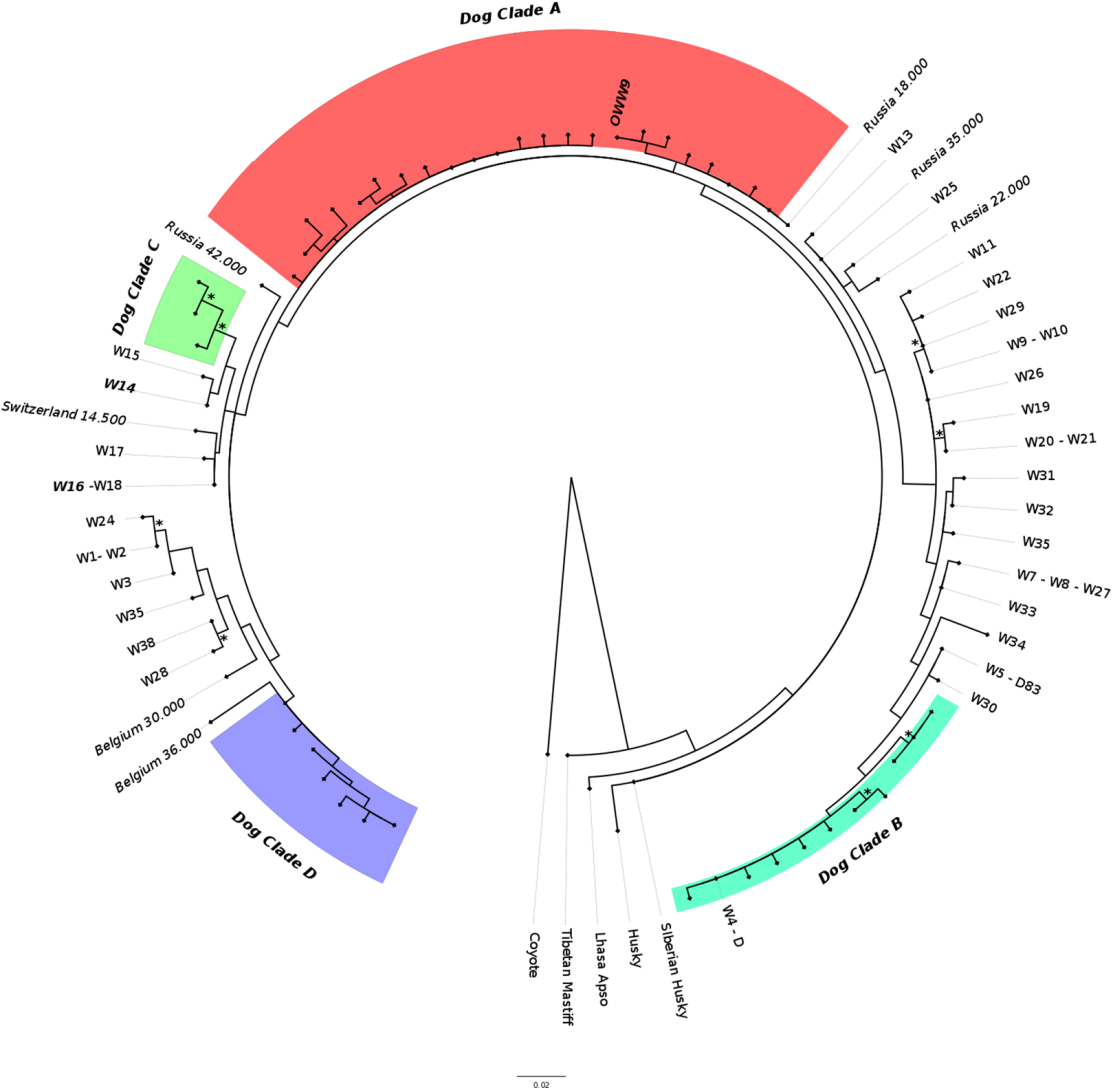
Maximum-likelihood tree based on the Alignment C (330 bp). Modern wolf haplotypes are represented by the letter W followed by a number and, in some cases, the letter D is placed next to the W to represent a terminal node where there is a shared haplotype between dogs and wolves. Ancient wolf samples are represented in the figure using their country of provenance and the reported age (in italics, BP). Dog Clades are highlighted as: Clade A (red); Clade B (turquoise); Clade C (green); Clade D (purple). Asterisks highlight statistical support when bootstrap values are found in >50% of 1,000 replicates. See Tables S2 and S3 for the list of samples.

The ML phylogenetic tree (Fig. 3) obtained from Alignment C, that included the single sample from this study (OWW9) successfully sequenced at the longer mtDNA fragment, showed a topology roughly similar to previously published trees for the clade that includes the Italian wolf population (Thalmann et al., 2013; Montana et al., 2017b). Although the support for the nodes was very low due to the limited alignment size, it could be noted that the contemporary Italian wolves, referable to haplotypes W14 and W16, belonged to a clade that comprises also haplotypes W15 (from Greece), W17 (from Croatia and Slovenia) and W18 (from Poland), and were closely related to another clade that includes a 14,500-years-old wolf sample from Switzerland (Thalmann et al., 2013) and ancient dogs. Interestingly, the OWW9 sample that, as already noted, shared the same haplotype of many modern dog breeds, fell completely outside the modern Italian wolf mtDNA (HVR1) lineage, close to dogs that belong to the canine clade A.

When we considered also the Bayesian analyses performed on the Alignment D (Fig. S1), in which we included the five ancient canid samples from Verginelli et al. (2005), together with the NJ network (Fig. 2), it was possible to observe an even higher genetic variability of the ancient Italian samples, distributed along the whole tree or sparse in the network. A total of 11 different haplotypes were detected in both studies, five from Verginelli and six from this study, but in none of them were found the two current Italian haplotypes. However, due to the morphological and genetic affinity to dogs of two samples (PIC4 and PIC5) from Verginelli et al. (2005)—which carried two different haplotypes—the total number of wolf haplotypes retrieved in both studies are nine.

Furthermore, most of the samples analyzed in this study are placed in close proximity to the modern Italian haplotypes (Fig. 2), with the only exception of the samples OWW18 (associated to haplogroup 1), OWW4 and OWW9 (which carry a dog haplotype). From the Bayesian tree it was also possible to observe that the OWW9 sample and those analyzed by Verginelli et al. (2005) are scattered throughout the tree, also falling within the three dog clades A, B and C (Fig. S1).

## Discussion

### Past genetic variability of Italian wolves

The Italian wolf population represents a case of genetic uniqueness highlighted by several studies both on the mitochondrial (Thalmann et al., 2013; Ersmark et al., 2016; Montana et al., 2017a) and nuclear DNA (vonHoldt et al., 2011). At the mtDNA level, this population is the only remaining wolf population in Europe belonging exclusively to a haplogroup that was widespread both in central and western Europe for over 40,000 years (Pilot et al., 2010) and in North America until the LGM (Leonard et al., 2007).

In this study, by successfully analyzing the mtDNA control region of 11 ancient wolf samples, we contributed to describing the genetic make-up of the Italian Pleistocene wolf population. Our results highlight that the ancient variability of Italian wolves was higher than today, a scenario that could be compatible with the well-known population reduction which started during the LGM and heightened in the last few centuries (Lucchini, Galov & Randi, 2004; vonHoldt et al., 2011; Boggiano et al., 2013; Pilot et al., 2014; Montana et al., 2017a, 2017b). Furthermore, by comparing the results from this study and the ones from Verginelli et al. (2005) we found no correspondence between the haplotypes detected in each study. From a total of 14 ancient Italian samples analyzed (excluding PIC4 and PIC5 from Verginelli, because morphologically and genetically ascribed to dogs), nine different wolf haplotypes were recovered from both studies.

All the haplotypes from the Late Pleistocene specimens here analyzed belong to haplogroup Hg2, and all of them except one correspond to haplotypes found in other ancient wolf samples from North America and Eurasia dating to between 47,700 and 16,000 years ago. Furthermore, most of the Pleistocene samples we analyzed are placed in close proximity to the current Italian haplotypes, with the only exception of the one carried by OWW4 and OWW9 that falls within the dog variability.

Moving from the Pleistocene to the Holocene specimens, a change in the haplotype occurrence is notable: the three samples here analyzed only match recent or extant haplotypes. Our findings could suggest a progressive diversity loss around the Pleistocene-Holocene transition testified by the only presence of modern haplotypes in the Holocene samples, which is compatible with the general trend of demographic contraction started in the Late Pleistocene *Canis lupus* populations (Vila et al., 1999; Verginelli et al., 2005) and confirmed also by genomic demographic inferences (Freedman et al., 2014; Fan et al., 2016).

In our study only one Holocene specimen, dated to 3,250 years ago and belonged to haplogroup Hg1. Therefore, the absence of any Hg1 haplotype in the Pleistocene samples, which is very unlikely to be observed only by chance in the set of samples analyzed, confirm that wolves with this haplogroup might have arrived in southern Europe more recently, confirming previous results (Pilot et al., 2010). Of course, it cannot be excluded that wolves belonging to Hg 1 inhabited certain geographical areas of Europe during the Pleistocene which have not yet been subjected to investigations. In any case, our results push back previous findings, which attested the presence of Hg1 wolves in Europe only in the last 1,400 years (Pilot et al., 2010), with the exception of an older sample from Italy dated to 9,800 years ago (PIC2) found to carry a Hg1 haplotype also present in two ancient dog samples (Fig. 2) (Leonard et al., 2002; Frantz et al., 2016) and which was previously included in a dog-dominated clade (Verginelli et al., 2005). Interestingly, the Hg1 haplotype carried by OWW18 matches one found in the extant Iberian wolf population, possibly representing the same migratory wave that might have largely replaced Hg2 wolves throughout Europe (Pilot et al., 2010). The lower presence of Hg1 wolves was possibly due to a higher geographical isolation of the Italian peninsula compared to other former *refugia*, which, combined with a possibly stronger genetic drift and historical bottleneck, could help to explain the higher genetic differentiation of Italian wolves compared to other populations (vonHoldt et al., 2010; Pilot et al., 2014; Galaverni et al., 2016; Montana et al., 2017a).

The two extant and unique Italian wolf haplotypes were not found among the set of analyzed ancient samples in Eurasia so far. Considering the higher variability retrieved in this area in the past, it might be possible that the current Italian haplotypes could have been already present in the genetic pool of the Pleistocene wolf population, but probably at frequencies too low to be sampled in the limited set and geographic area of ancient samples analyzed spanning also a wide chronological range. A second option could be that the Hg2 wolves isolated in the Italian glacial *refugia* experienced a turnover within their haplogroup as a result of in situ mutations, since they differ by only one or two mutations from all Hg2 haplotypes found in our ancient samples. Conversely, we are inclined to exclude the additional hypothesis of a complete replacement of haplotypes in the Italian population, because of the strong genetic similarity between ancient and current Italian haplotypes highlighted in our network analysis (Fig. 1 and Fig. 2) and in recent studies based on the mtDNA (Thalmann et al., 2013; Skoglund et al., 2015; Montana et al., 2017a). Therefore, we would explain this scenario by combination of limited sampling and in situ mutations, but further studies will be required to discriminate with certainty between these hypotheses.

### The canine haplotype of two Late Pleistocene canid samples

Surprisingly, the mtDNA haplotype of two samples (OWW4 and OWW9) from the archaeological site of Cava Filo corresponded to ancient (Stiller et al., 2006; Zhilin et al., 2014) and modern dogs. In particular, OWW9 was the only sample for which we obtained a longer mtDNA fragment and that we directly radiocarbon-dated in this study, obtaining an interval comprised between 25,008 and 24,409 years ago (cal 2σ). It is worth mentioning that OWW9, whose haplotype falls outside the current and ancient variability of Italian wolves, comes from a stratigraphic level where evidence of attendance by Paleolithic hunters-gatherers was recently described (Nenzioni, Marchesini & Marvelli, 2018; Paronuzzi et al., 2018) (see also Supplemental Article S1). Human hunter-gatherers communities are known to be involved in the dog domestication process, probably started from a now-extinct wolf population at least 12,500 years ago (Thalmann et al., 2013; Freedman et al., 2014; Skoglund et al., 2015; Frantz et al., 2016). However, the number of independent domestication events, as well as their geographical location and timing, remains highly contentious (Frantz et al., 2016; Botigué et al., 2017). Claims have been made of dog domestication about 30,000 years ago (Germonpré, Lázničková-Galetová & Sablin, 2012) but the earliest archaeological canid remains positively confirmed as dogs are dated to 15,000 years in Europe and 12,500 years in East Asia (Larson et al., 2012; Pionnier-Capitan et al., 2011).

Currently, the worldwide sequence variants of modern and ancient dogs are split into four main phylogenetic groups (namely A, B, C and D) (Savolainen et al., 1997) and the occurrence of a Late Pleistocene wolf carrying a haplotype belonging to the ancient canine clade A (Thai, Chung & Tran, 2017) could add valuable information on the temporal and spatial origin of haplotypes which could have been one of the “source” wolf population from which dogs originated. Given that, OWW9 could represent one of the oldest specimens with such haplotype. If this will be confirmed by the analysis of its full mtDNA, it could represent one of the oldest evidence of clade A in a canid sample since, as far as we know, the presence of this lineage in Europe and the Near East was previously attested only 13,250 years ago in Israel (Pionnier-Capitan, 2010) and 9,670 years ago in Italy (Verginelli et al., 2005; Deguilloux et al., 2009). Furthermore, the presence of a clade A haplotype in a 24,700-years-old canid sample, more than 10,000 years before the oldest clade A specimen attested in Israel, represents an interesting instance given that, although most modern European dogs belong to clade A or B (with a predominance of the first, attested at frequencies of 64% and 22%, respectively), the majority of ancient European dogs so far analyzed belonged to clades C or D (63% and 20%, respectively) (Frantz et al., 2016).

We must also acknowledge that the haplotype carried by OWW9 was found also in two modern samples, an attested wolf *x* dog hybrid from Iran (Aghbolaghi et al., 2014) and a wolf from China (Ersmark et al., 2016), analyzed for 686 bp of the d-loop mitochondrial region that, without further analyses, we cannot exclude to be a hybrid itself.

Obviously, it is absolutely necessary to investigate longer mtDNA fragments and autosomal DNA markers on the OWW9 sample, to verify or not all these speculative hypotheses in the context of dog domestication (Pires et al., 2017).

## Conclusions

In conclusion, our study contributes to shed a clearer light on the origin and past genetic variability of the Italian wolf population. Moreover, it also allows to better infer the patterns of variability and gene flow across past and modern Eurasian wolf populations, and to better explain their relationships with the modern Italian wolves. We highlight that, in line with the well-known population reduction, common to several species, started during the LGM and heightened in the last few centuries due to the higher anthropic pressure, the ancient genetic variability of Italian wolves has drastically decreased until today. We also detected the presence of a very interesting dog haplotype in a 24,700-years-old sample that deserve to be further analyzed with genomic approaches. Moreover, our results showed that only one Holocene specimen, dated 3,250 years ago, belonged to the Hg1, allowing us to hypothesize that wolves with this haplogroup might have arrived in southern Europe more recently, backdating a previous theory on the ancient population dynamics of the Eurasian wolf (Pilot et al., 2010). However, the limited spatial-temporal sampling combined with the short fragments we analyzed from only a single uniparental marker, the mitochondrial DNA, did not allow our data to reflect the full complexity of these dynamics. Furthermore, we also acknowledge that the chronological range here investigated was very wide, spanning from 25,000 to 1,000 years ago.

Therefore, we advocate that a larger number of ancient canid samples, as well as more in-depth genomic information, such as those derived by complete mitogenomes, nuclear SNP arrays or whole nuclear genomes, should be analyzed in the future for a deeper comprehension of the evolutionary history of European wolf.

## Supplemental Information

10.7717/peerj.6424/supp-1 Supplemental Information 1 Table S1. List of specimens included in this study with dating information. Chronological ranges were established by several radiocarbon dating analyses or were inferred from stratigraphy and material culture. (1) Radiocarbon dating was performed on specimens of the upper and lower S.U. that contained the samples from 1966s excavations (Pasini, 1970; Paronuzzi et al., 2018); (2) radiocarbon dating was performed on *Bison priscus*bones of the same S.U. of the samples genetically analysed in this study (Paronuzzi et al., 2018); (3) radiocarbon dating was directly performed on wolf sample OWW9 and OWW19 (this study); (4) regarding Monterenzio Vecchio site, the dating is based on stratigraphic information and material culture (Guerra et al., 2010).

10.7717/peerj.6424/supp-2 Supplemental Information 2 Table S2. List of modern mtDNA sequences downloaded from GenBank (Montana et al. 2017b). For each sample accession number, country/breed, taxon, single ID, mtDNA haplotypes at control region (CR code), dog clade and presence in the alignments A, C and D are indicated.

10.7717/peerj.6424/supp-3 Supplemental Information 3 Table S3. List of ancient mtDNA sequences downloaded from GenBank. For each sample accession number, country, locality, sample ID, age, authors, year of the study and presence in the alignments B, C and D are indicated.

10.7717/peerj.6424/supp-4 Supplemental Information 4 Figure S1. Bayesian tree based on the Alignment D (239 bp). In order to distinguish the ancient Italian samples, the branches of the five samples from Verginelli and colleagues (2005) are highlighted in dark gold while the branch of OWW9 is highlighted in dark blue. Modern wolf haplotypes are represented by the letter W followed by a number and, in some cases, the letter D is placed next to the W to represent a terminal node where there is a shared haplotype between dogs and wolves. Ancient wolf samples are represented in the figure using their country origin and the reported age in years before present (BP). Dog Clades are highlighted as: Clade A (red); Clade B (turquoise); Clade C (green); Clade D (purple). Asterisks highlight the nodes with statistical support > 50%. The samples used are listed in the Table S2 and Table S3.

10.7717/peerj.6424/supp-5 Supplemental Information 5 Supplemental material. This file contains information regarding the archaeological sites, the radiocarbon dating, extraction and amplification protocols.

## Additional Information and Declarations

### Data Availability

The following information was supplied regarding data availability: Sequences were deposited at Genbank: MH085470; MH085471; MH085472; MH085473; MH085474; MH085475; MH085476; MH085477; MH085478; MH085479; MH593822.
