# Supplementary material for: Old wild wolves: ancient DNA survey unveils population dynamics in Late Pleistocene and Holocene Italian remains
Source: PeerJ. 2019 Mar 27;7:e6424. doi: 10.7717/peerj.6424 (PMC6441319; doi:10.7717/peerj.6424)
Supplement: Supplemental Information 1 — Chronological ranges were established by several radiocarbon dating analyses or were inferred from stratigraphy and material culture. (1) Radiocarbon dating was performed on specimens of the upper and lower S.U. that contained the samples from 1966s excavations (Pasini, 1970; Paronuzzi et al., 2018); (2) radiocarbon dating was performed on Bison priscusbones of the same S.U. of the samples genetically analysed in this study (Paronuzzi et al., 2018); (3) radiocarbon dating was directly performed on wolf sample OWW9 and OWW19 (this study); (4) regarding Monterenzio Vecchio site, the dating is based on stratigraphic information and material culture (Guerra et al., 2010). [file peerj-07-6424-s001.pdf]

Museum ID	Sample ID	Site	SU	Recovery date	ID sample C14 analysis	C14 age (not cal.) (YBP)	Date range (YBP)	Ref. dating
124	OWW1	Cava Filo	G-D	1966	R362; R363 (1)	15,150±150; 18,200±200	18,599-17,869 (cal 2σ); 22,285-21,306 (cal 2σ)	Pasini, 1970; Paronuzzi et al., 2018
547	OWW2	Cava Filo	G-D	1966	R362; R364 (1)	15,150±150; 18,200±200	18,599-17,869 (cal 2σ); 22,285-21,306 (cal 2σ)	Pasini, 1970; Paronuzzi et al., 2018
556	OWW3	Cava Filo	G-D	1966	R362; R365 (1)	15,150±150; 18,200±200	18,599-17,869 (cal 2σ); 22,285-21,306 (cal 2σ)	Pasini, 1970; Paronuzzi et al., 2018
557	OWW4	Cava Filo	G-D	1966	R362; R366 (1)	15,150±150; 18,200±200	18,599-17,869 (cal 2σ); 22,285-21,306 (cal 2σ)	Pasini, 1970; Paronuzzi et al., 2018
06-027	OWW5	Cava Filo	99	2006	GRA 52971; GRN 32576 (2)	20,010±75; 20,050±100	24,275-23,535 (cal 2σ); 24,340-23,569 (cal 2σ)	Paronuzzi et al., 2018
07-201	OWW6	Cava Filo	100	2007	GRA 52970 (2)	14,450±50	17,877 - 17,220 (cal 2σ)	Paronuzzi et al., 2018
08-057	OWW7	Cava Filo	100	2008	GRA 52970 (2)	14,450±50	17,877 - 17,220 (cal 2σ)	Paronuzzi et al., 2018
09-049	OWW8	Cava Filo	99	2009	GRA 52971; GRN 32576 (2)	20,010±75; 20,050±100	24,275-23,535 (cal 2σ); 24,340-23,569 (cal 2σ)	Paronuzzi et al., 2018
09-050	OWW9	Cava Filo	99	2009	**OWW9 (3)**	20,517 ± 63	**25,008 - 24,409 (cal 2σ)**	**This study**
09-072	OWW10	Cava Filo	99	2009	GRA 52971; GRN 32576 (2)	20,010±75; 20,050±100	24,275-23,535 (cal 2σ); 24,340-23,569 (cal 2σ)	Paronuzzi et al., 2018
11-018	OWW11	Cava Filo	99	2011	GRA 52971; GRN 32576 (2)	20,010±75; 20,050±100	24,275-23,535 (cal 2σ); 24,340-23,569 (cal 2σ)	Paronuzzi et al., 2018
11-035	OWW12	Cava Filo	100	2011	GRA 52970 (2)	14,450 ± 50	17,877 - 17,220 (cal 2σ)	Paronuzzi et al., 2018
11-055	OWW13	Cava Filo	99	2011	GRA 52971; GRN 32576 (2)	20,010±75; 20,050±100	24,275-23,535 (cal 2σ); 24,340-23,569 (cal 2σ)	Paronuzzi et al., 2018
11-083	OWW14	Cava Filo	99	2011	GRA 52971; GRN 32576 (2)	20,010±75; 20,050±100	24,275-23,535 (cal 2σ); 24,340-23,569 (cal 2σ)	Paronuzzi et al., 2018
11-089	OWW15	Cava Filo	99	2011	GRA 52971; GRN 32576 (2)	20,010±75; 20,050±100	24,275-23,535 (cal 2σ); 24,340-23,569 (cal 2σ)	Paronuzzi et al., 2018
11-108	OWW16	Cava Filo	100	2011	GRA 52970 (2)	14,450 ± 50	17,877 - 17,220 (cal 2σ)	Paronuzzi et al., 2018
MV 07 (M1)	OWW17	Monterenzio Vecchio	3077	2007	/	/	3,290-3,200 (4)	Guerra et al., 2010
MV 2005 (M2)	OWW18	Monterenzio Vecchio	3017	2005	/	/	3,290-3,200 (4)	Guerra et al., 2010
MSDP 348	OWW19	Po River	/	2017	**OWW19 (3)**	1,004 ± 45	**983 - 793 (cal 2σ)**	**This study**
