# Supplementary material for: Old wild wolves: ancient DNA survey unveils population dynamics in Late Pleistocene and Holocene Italian remains
Source: PeerJ. 2019 Mar 27;7:e6424. doi: 10.7717/peerj.6424 (PMC6441319; doi:10.7717/peerj.6424)
Supplement: Supplemental Information 2 — For each sample accession number, country/breed, taxon, single ID, mtDNA haplotypes at control region (CR code), dog clade and presence in the alignments A, C and D are indicated. [file peerj-07-6424-s002.pdf]

GenBank Acc. Number	Locality/Breed	Taxon	Single ID	CR code	Dog Clade	Alignment A (57bp)	Alignment C (330bp)	Alignment D (239bp)
AF115687	Bulgaria	Wolf	W1	W1	/	v	v	v
AF115688	Bulgaria	Wolf	W2	W2	/	v	v	v
AF115689	Slovenia, Croatia	Wolf	W3	W3	/	v	v	v
AF115690	Greece	Wolf/Dog	W4A	W4	/	v	v	v
KF661047	Ukraine	Wolf/Dog	W4B	W4	/	v	v	v
AF115691	Bulgaria, Greece	Wolf	W5	W5	/	v	v	v
AF115692	Estonia, Latvia, Poland, Finland, Russia	Wolf	W7	W7	/	v	v	v
AF115693	Finland, Russia	Wolf	W8	W8	/	v	v	v
AF115694	Croatia, Bulgaria, Poland	Wolf	W9	W9	/	v	v	v
KF661054	Croatia	Wolf	W10	W10	/	v	v	v
KF661055	Israel	Wolf	W11	W11	/	v	v	v
AF115697	Israel	Wolf	W12	W12	/	v	v	v
AF115698	Finland	Wolf	W13A	W13	/	v	v	v
AF115699	Italy	Wolf	W14A	W14	/	v	v	v
AF115699	Greece	Wolf	W14B	W14	/	v	v	v
AF115699	Greece	Wolf	W14C	W14	/	v	v	v
AF115700	Greece	Wolf	W15	W15	/	v	v	v
AF115701	Italy	Wolf	W16A	W16	/	v	v	v
AF115701	Croatia, Bulgaria, Russia	Wolf	W16B	W16	/	v	v	v
GU059555	Slovenia, Croatia	Wolf	W17	W17	/	v	v	v
FJ978013	Poland	Wolf	W18	W18	/	v	v	v
AF115702	Spain, Portugal	Wolf	W19	W19	/	v	v	v
AF115703	Spain, Portugal	Wolf	W20A	W20	/	v	v	v
AF338807	Spain, Portugal	Wolf	W21	W21	/	v	v	v
KY550011	Poland	Wolf	W22	W22	/	v	v	v
JX508635	Estonia, Latvia, Poland	Wolf	W23	W23	/	v	v	v
FJ978009	Estonia	Wolf	W24A	W24	/	v	v	v
FJ978009	Spain	Wolf	W24B	W24	/	v	v	v
DQ480506	Saudi Arabia	Wolf	W25	W25	/	v	v	v
KF661041	China	Wolf	W26	W26	/	v	v	v
KF661052	Sweden	Wolf	W27	W27	/	v	v	v
KF661051	Iran	Wolf	W28	W28	/	v	v	v
KF661043	India	Wolf	W29	W29	/	v	v	v
KF661056	Canada	Wolf	W30A	W30	/	v	v	v
KF661062	Canada	Wolf	W30B	W30	/	v	v	v
KF661074	Canada	Wolf	W31	W31	/	v	v	v
KF661066	Alaska	Wolf	W32	W32	/	v	v	v
KF661071	Alaska	Wolf	W33	W33	/	v	v	v
KF661058	Alaska	Wolf	W34	W34	/	v	v	v
KF661064	USA	Wolf	W35	W35	/	v	v	v
KF661060	Mexico	Wolf	W36	W36	/	v	v	v
KF661065	Mexico	Wolf	W37	W37	/	v	v	v
KF661050	Oman	Wolf	W38	W38	/	v	v	v
KP665919	Hungary	Wolf	W39	W39	/	/	/	/
AF115704	various dog breeds	Dog	D1A	D1	B	/	v	v
AF115704	Australian Terrier	Dog	D1B	D1	B	/	v	v
AF115705	Lupino del Gigante	Dog	D2	D2	B	/	v	v
KJ637145	Hybrid, various dog breeds	Dog/Wolf	D5-6A	D5-6	A	/	v	v
AY656751	Gordon Setter, Golden Retriever	Dog/Wolf	D5-6B	D5-6	A	/	v	v
AF115710	West Highland White Terrier	Dog	D8A	D8	A	/	v	v
AF115710	various dog breeds	Dog	D8B	D8	A	/	v	v
AF115710	French Bull Dog	Dog	D8C	D8	A	/	v	v
AF115711	Irish Setter	Dog	D9A	D9	A	/	v	v
AF115711	various dog breeds	Dog	D9B	D9	A	/	v	v
AF115711	Lupino del Gigante	Dog	D9C	D9	A	/	v	v
AF115712	Siberian Husky	Dog	D10	D10	A	/	v	v
AF115713	Various Terrier, Newfoundland	Dog	D11	D11	A	/	v	v
AF115714	English Springer Spaniel	Dog	D13A	D13	A	/	v	v
AF115714	various dog breed	Dog	D13B	D13	A	/	v	v
AF115715	Sapsaree	Dog	D14A	D14	A	/	v	v
AF115715	NA	Dog	D14B	D14	A	/	v	v
AF115715	Dachshund, Australian and German Shepherd	Dog	D14C	D14	A	/	v	v
DQ480502	Jamthund	Dog	D51	D51	D	/	v	v
DQ480492	Jamthund	Dog	D52	D52	D	/	v	v
JF342905	various dog breeds	Dog	D53A	D53	A	/	v	v
EU408302	Toy Poodle	Dog	D53B	D53	A	/	v	v
EU408305	Vizsla	Dog	D53C	D53	A	/	v	v
DQ480489	German Shepherd	Dog	D54	D54	C	/	v	v
KJ637135	various dog breed	Dog	D55	D55	B	/	v	v
DQ480501	Jamthund, Cocker Spaniel, Black Russ Terrier	Dog	D56A	D56	C	/	v	v
EU223397	Australian Sheperd	Dog	D56B	D56	C	/	v	v
EU408279	Havanese	Dog	D56C	D56	C	/	v	v
EU408291	Pomeranian	Dog	D56D	D56	C	/	v	v
EU408245	Akita	Dog	D57A	D57	A	/	v	v
EU408265	Welsh Corgi	Dog	D57B	D57	A	/	v	v
EU408282	Keenshond	Dog	D58A	D58	A	/	v	v
KJ637044	NA	Dog	D58B	D58	A	/	v	v
EU408288	Norwegian Elkhound	Dog	D59A	D59	D	/	v	v
JF342824	Norwegian Elkhound	Dog	D59B	D59	D	/	v	v
EU408293	PitBull Terrier	Dog	D60A	D60	C	/	v	v
JF342821	Dogue de Bordeaux	Dog	D60B	D60	C	/	v	v
JF342816	Basenji	Dog	D61	D61	A	/	v	v

JF342838	Akita	Dog	D62	D62	B	/	v	v
JF342839	Bouvier Des Flandres	Dog	D63A	D63	C	/	v	v
KF907309	German Shepherd	Dog	D63B	D63	C	/	v	v
JF342887	d, Nova Scotia Duck Trolling Retriever, English Co	Dog	D64	D64	C	/	v	v
AB499816	Kishu	Dog	D65	D65	A	/	v	v
AB499817	Siberian Husky	Dog	D66	D66	A	/	v	v
AY656742	Old English Sheepdog (Bobtail)	Dog	D67	D67	A	/	v	v
AY656745	English Springer Spaniel	Dog	D68	D68	B	/	v	v
AY656752	Standard Schnauzer	Dog	D69	D69	B	/	v	v
AY656754	Chinese Crested	Dog	D70	D70	A	/	v	v
EU408258	Cockapoo	Dog	D71	D71	B	/	v	v
EU408262	Chihuahua	Dog	D72	D72	A	/	v	v
EU408268	Cocker Spanie, German Shepherd	Dog	D73	D73	B	/	v	v
EU408300	Tibetan Mastiff	Dog	D74	D74	A	/	v	v
EU408303	NA	Dog	D75	D75	B	/	v	v
FJ817364	Golden Retriever	Dog	D76	D76	B	/	v	v
JF342810	Bichon Frise	Dog	D77	D77	A	/	v	v
JF342815	Catahoula Leopard Dog	Dog	D78	D78	B	/	v	v
JF342817	Plott Hound	Dog	D79	D79	A	/	v	v
JF342818	Boston Terrier	Dog	D80	D80	C	/	v	v
JF342822	Neopolitan Mastiff	Dog	D81	D81	A	/	v	v
JF342823	Irish Wolfhound, Pyrenean Mastiff	Dog	D82	D82	A	/	v	v
JF342826	Japanese Chin	Dog	D83	D83	B	/	v	v
JF342836	Bloodhound	Dog	D84	D84	A	/	v	v
JF342852	Lhasa Apso	Dog	D85	D85	A	/	v	v
JF342859	Afghan Hound	Dog	D86	D86	D	/	v	v
JF342862	Tibetan Mastiff	Dog	D87	D87	A	/	v	v
JF342864	Bichon Frise	Dog	D88	D88	B	/	v	v
JF342868	English Shepard	Dog	D89	D89	C	/	v	v
JF342903	Chow Chow	Dog	D90	D90	A	/	v	v
KJ637041	NA	Dog	D91	D91	A	/	v	v
KJ637043	NA	Dog	D92	D92	A	/	v	v
KJ637048	NA	Dog	D93	D93	A	/	v	v
KJ637069	NA	Dog	D94	D94	A	/	v	v
KJ637087	NA	Dog	D95	D95	A	/	v	v
KJ637104	NA	Dog	D96	D96	B	/	v	v
KJ637107	NA	Dog	D97	D97	B	/	v	v
KJ637109	NA	Dog	D98	D98	B	/	v	v
KJ637138	NA	Dog	D99	D99	C	/	v	v
KJ637142	NA	Dog	D100	D100	C	/	v	v
KJ789955	NA	Dog	D101	D101	A	/	v	v
KM113774	NA	Dog	D102	D102	A	/	v	v
KJ139080	NA	Dog	D103	D103	NA	/	/	/
HQ287728	NA	Dog	D104	D104	NA	/	/	/
AY656737	Basenji	Dog	Basenji	Basenji	A	/	v	v
