# Supplementary material for: Old wild wolves: ancient DNA survey unveils population dynamics in Late Pleistocene and Holocene Italian remains
Source: PeerJ. 2019 Mar 27;7:e6424. doi: 10.7717/peerj.6424 (PMC6441319; doi:10.7717/peerj.6424)
Supplement: Supplemental Information 3 — For each sample accession number, country, locality, sample ID, age, authors, year of the study and presence in the alignments B, C and D are indicated. [file peerj-07-6424-s003.pdf]

GenBank Acc. Number	Country	Locality	Sample ID	Age (BP)	Author and year of the study	Alignment B (57 bp)	Alignment C (330bp)	Alignment D (239bp)
MH593822	Italy	Cava Filo	OWW4	22,285 - 17,869	This study	v		
MH085470	Italy	Cava Filo	OWW8	23,940	This study	v		
MH085471	Italy	Cava Filo	OWW9	24,700	This study	v	v	v
MH085472	Italy	Cava Filo	OWW11	23,940	This study	v		
MH085473	Italy	Cava Filo	OWW12	17,550	This study	v		
MH085474	Italy	Cava Filo	OWW13	23,940	This study	v		
MH085475	Italy	Cava Filo	OWW15	23,940	This study	v		
MH085476	Italy	Cava Filo	OWW16	17,550	This study	v		
MH085477	Italy	Monterenzio Vecchio	OWW17	3,250	This study	v		
MH085478	Italy	Monterenzio Vecchio	OWW18	3,250	This study	v		
MH085479	Italy	Po River	OWW19	890	This study	v		
AY163878.1	Bolivia	Iwawi	JAL 330	>1,000	Leonard 2002	v		
AY163879.1	Bolivia	Iwawi	JAL 331	>1,000	Leonard 2002	v		
AY163880.1	Bolivia	Iwawi	JAL 332	>1,000	Leonard 2002	v		
AY163881.1	Bolivia	Iwawi	JAL 334	>1,000	Leonard 2002	v		
AY163882.1	Bolivia	Iwawi	JAL 337	>1,000	Leonard 2002	v		
AY163883.1	Peru	Chiribaja Baja	JAL 365	1,000	Leonard 2002	v		
AY163884.1	Peru	Chiribaja Baja	PC5	1,000	Leonard 2002	v		
AY163885.1	Peru	Chiribaja Baja	PC6	1,000	Leonard 2002	v		
AY163886.1	Mexico	Teotihuacan	PC8	1,300	Leonard 2002	v		
AY163887.1	Mexico	Texcoco	PC10	800	Leonard 2002	v		
AY163888.1	Mexico	Tula	PC12	1,400	Leonard 2002	v		
AY163889.1	Mexico	Tula	PC13	1,400	Leonard 2002	v		
AY163890.1	Mexico	Tula	PC14	1,400	Leonard 2002	v		
AY741666.1	Italy	Latium(Palidoro)	PIC1	14,670 ±130	Verginelli 2005	v		v
AY741667.1	Italy	Apulia (Romanelli Cave)	PIC2	9,860±50	Verginelli 2005	v		v
AY741668.1	Italy	Apulia (Romanelli Cave)	PIC3	9,670±40	Verginelli 2005	v		v
AY741669.1	Italy	Latium (Casal del Dolce)	PIC4	4,110±40	Verginelli 2005	v		v
AY741670.1	Italy	Latium (Vejano)	PIC5	3,040±40	Verginelli 2005	v		v
DQ852634.1	Czech Republic	Vypustek	6	24800	Stiller 2006	v		
DQ852635.1	Czech Republic	Vypustek	11	47700	Stiller 2006	v		
DQ852636.1	Czech Republic	Slouper cave	14	45200	Stiller 2006	v		
DQ852661.1	Ukraine	Zaskalnaya-9	17	32000	Stiller 2006	v		
DQ852662.1	Ukraine	Nerubajskoe-4	18	33800	Stiller 2006	v		
DQ852637.1	Russia	Zamyatino-7;Lipetsk Province	20	1100-2800	Stiller 2006	v		
DQ852638.1	Russia	Tronny grotto;Sakhalin	21	18300	Stiller 2006	v		
DQ852640.1	Russia	Razboinichya cave; Altai	26	37000	Stiller 2006	v		
DQ852641.1	Russia	Razboinichya cave; Altai	28	52200	Stiller 2006	v		
DQ852642.1	Russia	Razboinichya cave; Altai	29	>54700	Stiller 2006	v		
DQ852643.1	Germany	Roxdorf Frankenthal	32	1700	Stiller 2006	v		
DQ852644.1	Belgium	Goyet cave A1	33	25000-15000	Stiller 2006	v		
DQ852645.1	Belgium	Goyet cave A1	34	25000-15000	Stiller 2006	v		
DQ852646.1	Belgium	Goyet cave A3	36	30000-20000	Stiller 2006	v		
DQ852647.1	Belgium	Goyet cave A4	37	30000-20000	Stiller 2006	v		
DQ852648.1	Belgium	Goyet cave A1	38	16800	Stiller 2006	v		
DQ852649.1	Belgium	Goyet cave B4	42	29800	Stiller 2006	v		
DQ852650.1	Belgium	Furfooz,Trou des Nutons	44	26200	Stiller 2006	v		
DQ852651.1	Germany	Eich am Rhein	45	2000	Stiller 2006	v		
DQ852652.1	Germany	Eich am Rhein	47	2000	Stiller 2006	v		
DQ852653.1	Germany	Ginsheim	48	32200	Stiller 2006	v		
DQ852654.1	Germany	Eich am Rhein	49	1700	Stiller 2006	v		
DQ852655.1	Germany	Eich am Rhein	50	1900	Stiller 2006	v		
DQ852656.1	Germany	Eich am Rhein	51	2000	Stiller 2006	v		
DQ852657.1	Germany	Eich am Rhein	52	1900	Stiller 2006	v		
DQ852658.1	Germany	Eich am Rhein	53	1300	Stiller 2006	v		
DQ852659.1	Germany	Eich am Rhein	54	2200	Stiller 2006	v		
DQ852660.1	Hungary	Istallosko cave	61	39700	Stiller 2006	v		
/	Alaska	Eastern Beringia	PW1	20,305 ± 385	Leonard 2007	v		
/	Alaska	Eastern Beringia	PW2	>47,170	Leonard 2007	v		
/	Alaska	Eastern Beringia	PW3	>38,570	Leonard 2007	v		
/	Alaska	Eastern Beringia	PW4	17,640 ±240	Leonard 2007	v		
/	Alaska	Eastern Beringia	PW5	17,330 ± 290	Leonard 2007	v		
/	Alaska	Eastern Beringia	PW6	14,030 ± 50	Leonard 2007	v		
/	Alaska	Eastern Beringia	PW7	20,920 ± 70	Leonard 2007	v		
/	Alaska	Eastern Beringia	PW8	18,380 ± 390	Leonard 2007	v		
/	Alaska	Eastern Beringia	PW9	15,870 ± 190	Leonard 2007	v		
/	Alaska	Eastern Beringia	PW10	12,600 ± 150	Leonard 2007	v		
/	Alaska	Eastern Beringia	PW11	21,490 ± 110	Leonard 2007	v		
/	Alaska	Eastern Beringia	PW12	16,800 ± 210	Leonard 2007	v		
/	Alaska	Eastern Beringia	PW13	28,610 ± 860	Leonard 2007	v		
/	Alaska	Eastern Beringia	PW14	>32,100	Leonard 2007	v		
/	Alaska	Eastern Beringia	PW15	>39,200	Leonard 2007	v		
/	Alaska	Eastern Beringia	PW16	37,733 ± 2633	Leonard 2007	v		
EU287462	France	Villeneuve-Tolosane	VTC3	5950-5750	Deguilloux 2009	v		
EU287461	France	Villeneuve-Tolosane	VTC2	5950-5751	Deguilloux 2009	v		
EU287460	France	Villeneuve-Tolosane	VTC1	5950-5752	Deguilloux 2009	v		
KF661079	Belgium	Goye niveau 4	Belgium 36k	36,000	Thalman 2013	v	v	v
KF661080	Belgium	Goye niveau 4	Belgium 30k	30,000	Thalman 2013	v	v	v
KF661081	Russia	Medvezya cave	Russia 18k	18,000	Thalman 2013	v	v	v
KF661083	USA	Koster site, Illinois	USA 8.5k	8,500	Thalman 2013	v		
KF661084	Argentina	Cerro Lutz	Argentina 1k	1,000	Thalman 2013	v		

KF661085	Russia	Kostenki 4	Russia 22k	22,000	Thalman 2013	v	v	v
KF661086	USA	Florida	USA 1k	1,000	Thalman 2013	v		
KF661087	Switzerland	Kesslerloch cave	Switz1 14.5k	14,500	Thalman 2013	v		
KF661088	Alaska	Eastern Beringia	Alaska 28k	28,000	Thalman 2013	v		
KF661090	Alaska	Eastern Beringia	Alaska 20.8k	20,800	Thalman 2013	v		
KF661091	Switzerland	Kesslerloch cave	Switz2 14.5k	14,500	Thalman 2013	v		
KF661092	Russia	Razboinichya cave; Altai	Russia 33.5k	33,500	Thalman 2013	v	v	v
KF661094	Germany	Kartstein cave	Germany 12.5k	12,500	Thalman 2013	v		
LM993795.1	Russia	Urals (Beregovaya 2)	dog coprolite	9,500	Zhilin 2014	v		
JX173682.1	Russia	Razboinichya cave; Altai	CAN1	33,000	Druzhkova 2013	v		
KJ909859.1	Russia	Aachim, East Siberian Sea Coast	S603	1,760 ± 40	Lee 2015	v		
KJ909858.1	Russia	Aachim, East Siberian Sea Coast	S502	1,740 ± 40	Lee 2015	v		
KJ909864.1	Russia	DuvanyYar, Lower Kolyma River	S503	>47,000	Lee 2015	v		
KJ909863.1	Russia	Zhokhov Island, New Siberian Islands	S602	8,710 ± 50	Lee 2015	v		
KJ909862.1	Russia	DuvanyYar, Lower Kolyma River	S504	>47,000	Lee 2015	v		
KJ909856.1	Russia	Yana RHS, Lower Yana River	S601	27,840 ± 220	Lee 2015	v		
KJ909851.1	Russia	Yana RHS, Lower Yana River	S501	28,520 ± 240	Lee 2015	v		
/	Hungary	Alsónyek-Bátaszék	aEurA01	6,315	Frantz 2016	v		
/	Hungary	Alsónyek-Bátaszék	aEurA02	6,315	Frantz 2016	v		
/	Hungary	Alsónyek-Bátaszék	aEurA03	6,315	Frantz 2016	v		
/	Hungary	Alsónyek-Bátaszék	aEurA04	6,315	Frantz 2016	v		
/	Hungary	Alsónyek-Bátaszék	aEurA05	6,315	Frantz 2016	v		
/	Hungary	Alsónyek-Bátaszék	aEurA06	6,315	Frantz 2016	v		
/	Hungary	Alsónyek-Bátaszék	aEurA07	6,315	Frantz 2016	v		
/	Hungary	Alsónyek-Bátaszék	aEurA17	6,315	Frantz 2016	v		
/	Hungary	Alsónyek-Bátaszék	aEurA18	6,315	Frantz 2016	v		
/	Hungary	Alsónyek-Bátaszék	aEurA19	6,315	Frantz 2016	v		
/	Indonesia	NA	aOcea20	3,000	Frantz 2016	v		
/	Indonesia	NA	aOcea21	2,545	Frantz 2016	v		
/	New Zealand	NA	aOcea22	3,000	Frantz 2016	v		
/	Vietnam	NA	aOcea32	3,700	Frantz 2016	v		
/	Vietnam	NA	aOcea33	3,200	Frantz 2016	v		
/	Antigua	Indian Creek Site	aAm17	1,650	Frantz 2016	v		
/	Antigua	Indian Creek Site	aAm18	950	Frantz 2016	v		
/	USA	Morel	aAm36	1,960	Frantz 2016	v		
KX898307	Russia	Siberia-Taimyr	SW047	35,000	Ersmark et al 2016	v		
KX898308	Russia	Siberia-Taimyr	TX034	42,000	Ersmark et al 2016	v	v	v
KX898331	Greenland	Pearyland	ZMK 112a/1950	1,480	Ersmark et al 2016	v		
