# Supplementary figures and images for: Old wild wolves: ancient DNA survey unveils population dynamics in Late Pleistocene and Holocene Italian remains
Source: PeerJ. 2019 Mar 27;7:e6424. doi: 10.7717/peerj.6424 (PMC6441319; doi:10.7717/peerj.6424)

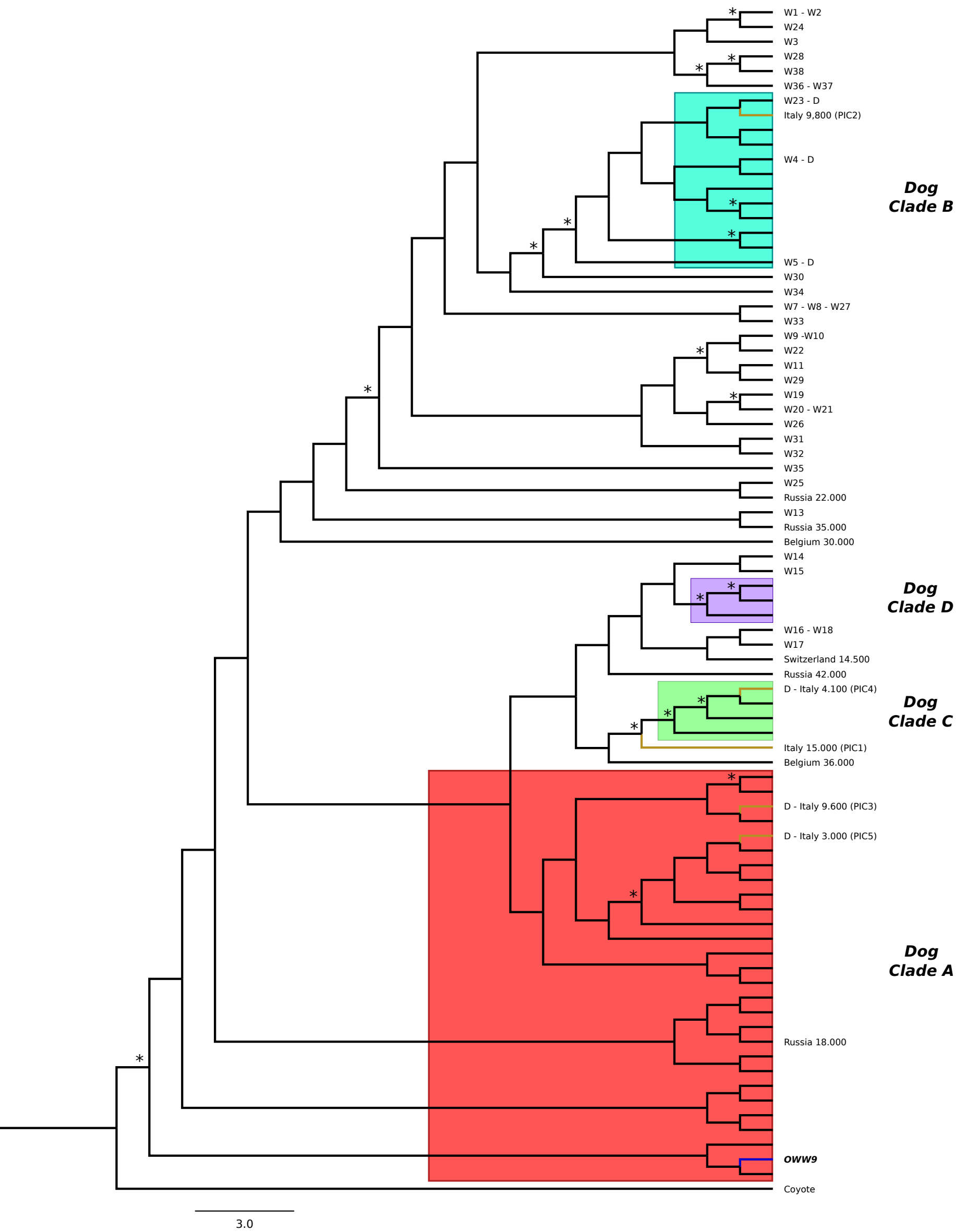

Supplement: Supplemental Information 4 — In order to distinguish the ancient Italian samples, the branches of the five samples from Verginelli and colleagues (2005) are highlighted in dark gold while the branch of OWW9 is highlighted in dark blue. Modern wolf haplotypes are represented by the letter W followed by a number and, in some cases, the letter D is placed next to the W to represent a terminal node where there is a shared haplotype between dogs and wolves. Ancient wolf samples are represented in the figure using their country origin and the reported age in years before present (BP). Dog Clades are highlighted as: Clade A (red); Clade B (turquoise); Clade C (green); Clade D (purple). Asterisks highlight the nodes with statistical support > 50%. The samples used are listed in the Table S2 and Table S3. [file peerj-07-6424-s004.pdf]
