# Supplementary material for: Old wild wolves: ancient DNA survey unveils population dynamics in Late Pleistocene and Holocene Italian remains
Source: PeerJ. 2019 Mar 27;7:e6424. doi: 10.7717/peerj.6424 (PMC6441319; doi:10.7717/peerj.6424)
Supplement: Supplemental Information 5 — This file contains information regarding the archaeological sites, the radiocarbon dating, extraction and amplification protocols. [file peerj-07-6424-s005.pdf]

# SUPPLEMENTAL MATERIAL

## Old wild wolves: ancient DNA survey unveils population dynamics in Late Pleistocene and Holocene Italian remains

**Authors:**

Marta Maria Ciucani[1–2], Davide Palumbo[3], Marco Galaverni[4–5], Patrizia Serventi[1–6], Elena Fabbri[5], Gloria Ravegnini[7], Sabrina Angelini[7], Elena Maini[8], Davide Persico[9], Romolo Caniglia[5], Elisabetta Cilli[1]

**Affiliations**:

[1] Laboratories of Physical Anthropology and Ancient DNA, Department of Cultural Heritage, University of Bologna, Ravenna Campus, Italy;

[2] Natural History Museum of Denmark, 1350 Copenhagen, Denmark;

[3] Ente di Gestione per i Parchi e la Biodiversità Emilia Orientale, Italy;

[4] WWF Italia, Conservation Unit, Rome, Italy;

[5]Area per la Genetica della Conservazione BIO-CGE of the Italian Institute for Environmental Protection and Research (ISPRA), Ozzano dell'Emilia, Bologna, Italy;

[6] Department of Biological, Geological & Environmental Sciences - BiGeA, University of Bologna, Italy;

[7] Department of Pharmacy and Biotechnology, University of Bologna, Bologna, Italy;

[8] ArcheoLaBio - Research Centre for Bioarchaeology, Department of History and Culture, University of Bologna, Ravenna, Italy;

[9] Department of Chemistry, Life Sciences and Environmental Sustainability, University of Parma, Italy.

## Archaeological sites description

The 19 canid samples analysed in this study were retrieved from three different archaeological sites located in northern Italy (Fig. S2 and Fig. S3), spanning from Late Pleistocene to Holocene.

All the samples were selected based on the well-defined archaeological contexts or radiocarbon dating was carried out to provide further support of their ages (Table 1 and Table S1).

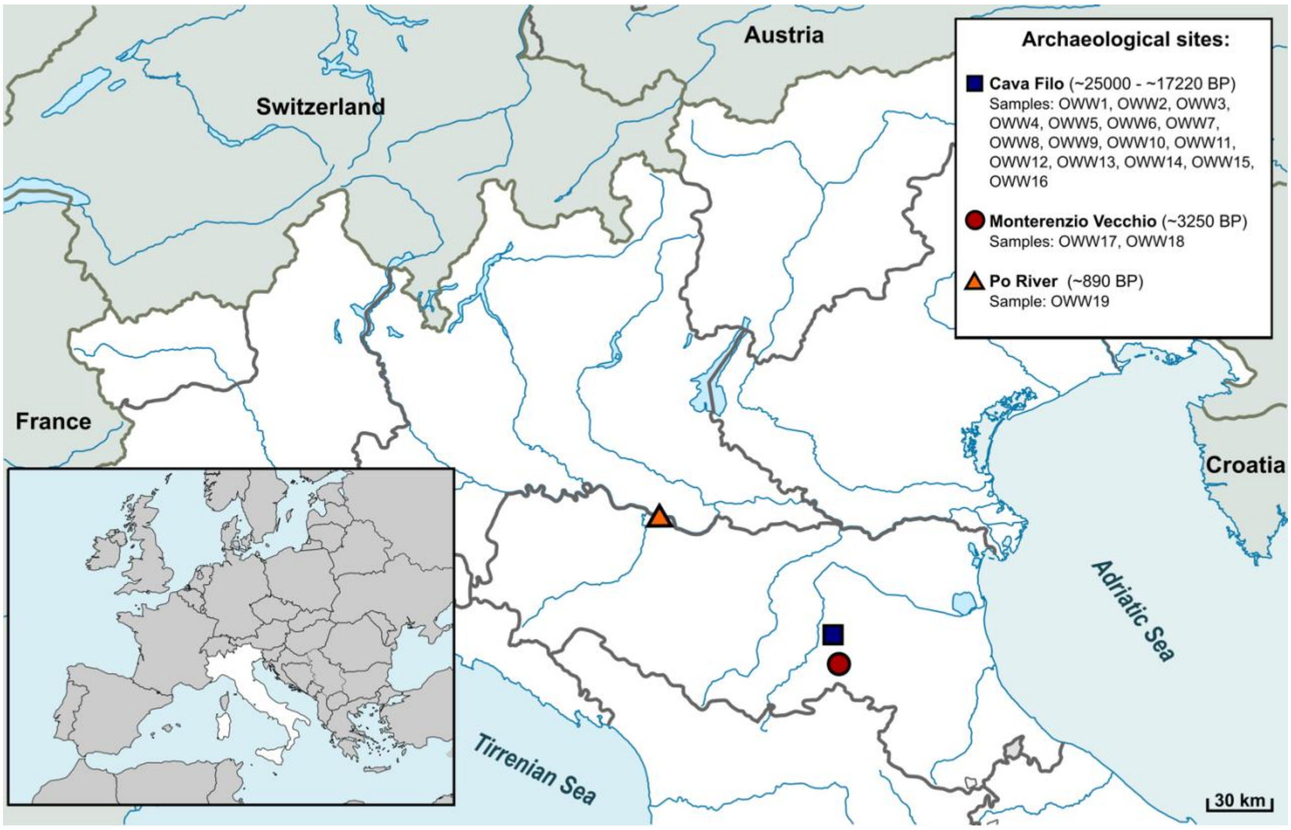


Figure S2. Geographic locations of the sites where the analysed samples were collected.

The oldest site of Cava Filo is represented by a blue square, Monterenzio Vecchio and Po River sites are shown by a red circle and an orange triangle respectively.

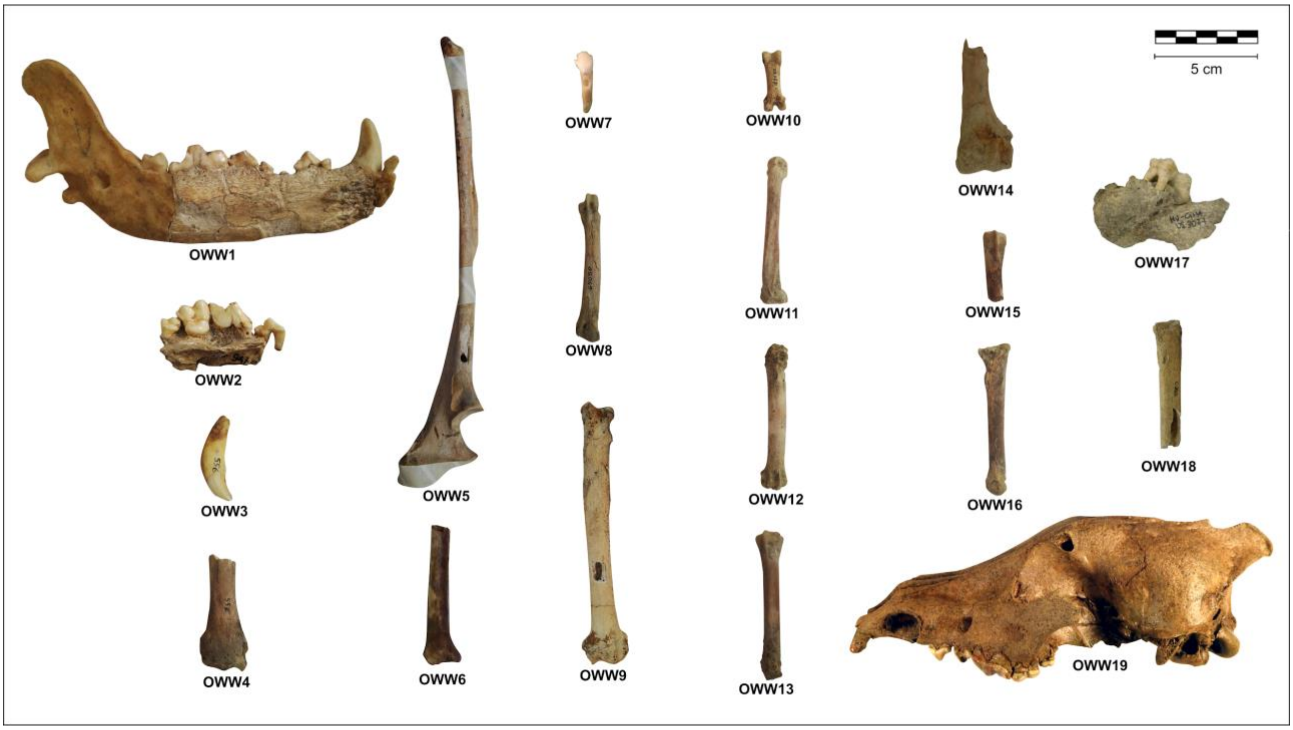


Figure S3. Samples analysed in this study. The code OWW followed by the number 1 to 16 is referred to the specimens excavated in the Cava Filo site, while OWW17 and OWW18 are referred to Monterenzio Vecchio site and OWW19 is the sample found along the Po River.

**1. Cava Filo site**

The site of Cava Filo (225 meters above mean sea level, AMSL) (Fig. S2) is a sedimentary context of a karst system constituted by relic fluvio-karst galleries originated in the Late Pleistocene. Several species, of which the large part closely related to cold climate, has been recovered so far, such as *Bison priscus*, *Megaloceros giganteus*, *Capreolus capreolus*, *Canis lupus*, *Lepus timidus* and also micromammals such as *Microtus arvalis*, *Chionomys nivalis* and *Marmota marmota*. *Canis lupus* is the only carnivore recovered in this site. Thanks to the rich paleontological deposit and the abundant palynological evidence contained in the cavities present inside the gypsum, the site constitutes a reference for the reconstruction of stratigraphy and paleoenvironment of the Italian Late Pleistocene, in particular of the period corresponding to the Last Glacial Maximum (Paronuzzi *et al.*, 2018).

The presence of a pitfall is interesting, as it probably represented a trap for the animals attracted by the water. In this peculiar geological-geomorphological context, the presence of water, the closed depression and the karst sinkhole represented certainly a favourable topographic-environmental situation for the hunting of various animal species, such as *Bison priscus*, by men and predators like wolves. The C14 dating performed in the different stratigraphic units (S.U.) of this site (see below and Table 1) attributed the faunal remains of this site in a chronological range comprised between

25,000 and 17,220 years old. *Bison priscus* was the most abundant representative of the faunal assemblages recovered and constitute the 73% of the remains of macromammals. Contrary to what one might expect from such a context, the second for abundance was *Canis lupus* (more than 13% of the recovered bones), followed by roe deer (*Capreolus capreolus*) and *Megaloceros*. Several bones showed signs of the predation by medium-large carnivores, probably wolves, but a bison tibia exhibits a very interesting evidence of cut marks, represented by two tidy linear and parallel V-shaped lines in the proximity of the popliteal muscle. For those peculiar and clear characteristics, these marks were attributed to anthropic signs on the bone surface, left by a lithic tool during the animal slaughtering. C14 dating on a remain of this S.U. provide a calibrated age of ~ 24,000 years BP (Paronuzzi *et al.*, 2018).

The extraordinary abundance of the *Bison priscus* remains and the significant recovery of the marks in this tibia, coupled with the numerous traces of anthropic attendance in the adjacent karst area, constitute important evidence that support the presence of specialized groups of Upper Paleolithic hunters, that future surveys will better clarify, especially for this site (Nenzioni, Marchesini and Marvelli, 2018). The relative abundance of the findings belonging to wolves could describe a context in which the canids were used to scavenge the remains of hunted ungulates, entering in frequent contact with hunter-gatherers, but at the current state of knowledge, this hypothesis remains highly speculative.

The selection of the specimens included in this study was based on both the S.U., the year of archaeological excavation and the characteristic of the samples: teeth and compact bones were preferred rather than spongy bones.

***Radiocarbon Dating Analysis of Cava Filo samples***

All samples here analysed were from well-defined paleontological contexts for which *Bison* remains (coming from the same S.U. of the canid samples analysed in this study) were previously radiocarbon dated in Rome and at the University of Groningen Centre for Isotope Research (Table S1) (Pasini, 1969, 1970; Nenzioni *et al.*, 2018; Paronuzzi *et al.*, 2018). In particular, radiocarbon dating was performed: i) for samples OWW 1, 2, 3, 4 in Rome on specimens of the upper and lower SU that contained the wolf samples from 1966s excavations (Pasini, 1970; Paronuzzi *et al.*, 2018); ii) for samples OWW 5, 6, 7, 8 ,10, 11, 12, 13, 14, 15, 16 at the University of Groningen Centre for Isotope Research on *Bison priscus* bones of the same S.U. of the samples genetically analysed in this study (Paronuzzi *et al.*, 2018) (See also Table S1). Moreover, one sample analysed in this study (OWW9), particularly interesting for its haplotype assignment to the canine clade A, was sent for

radiocarbon dating at the Laboratory of Ion Beam Physics of ETH, Zurich, to date it directly and provide further support of its age (Fig. S4 and Table S1).

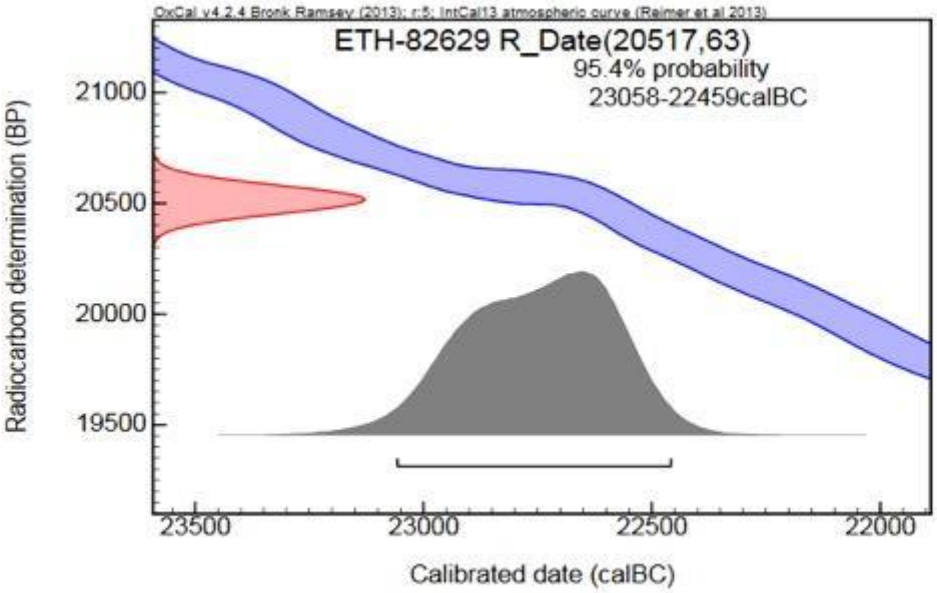


Figure S4. Calibration curve of the radiocarbon age of the OWW9 sample which, with a probability of 95.4%, it was assigned to a chronological range of 23,058-22,459 BC corresponding to 25,008 - 24,409 years ago.

**2. Monterenzio Vecchio site**

The upland site of Monterenzio Vecchio (Fig. S2 and Fig. S5) is located in Emilia-Romagna Region of Northern Italy, about 600 meters AMSL on the homonymous mountain in the Tuscan-Emilian Apennines and a few kilometres from the border with Tuscany. The archaeological excavations investigated an area of 450 square meters and resulted in the identification of three consequent occupation phases, all dating to the Late Bronze Age (ca. 3,290-3,200 BP). Each phase was built upon an artificial terracing made using displaced earth fillings contained within structures made with medium-sized stones, eventually levelled and stabilized with finer soil. The earliest levels (Phase I) were quite well preserved and included a large floor characterized by a massive presence of anthropogenic materials (ceramic, bones, charcoal fragments, etc.), numerous post-holes and larger holes, and a small dump area where potsherds, burned and unburned animal bones and tools made from animal bones and deer antler have been found (Guerra *et al.*, 2010).

The archaeozoological study revealed an animal economy based mainly on the exploitation of domestic animals (96%). However, the faunal sample included also different wild species hunted

for their meat, such as wild boar, deer and roe deer, but also wolves and bears as the possible result of targeted hunts with defensive purposes and/or for obtaining their fur (Maini, 2012).
From this archaeological site we selected two samples (OWW17 and OWW18), respectively a third left metatarsal and an upper right jaw bone with a premolar tooth (Fig. S3) (Guerra et al., 2010, Maini, 2012). Despite the fact that the fragmentation degree of this remains prevents taking the canonical measurements necessary for a species discrimination (Driesch von Den, 1976), the two osteological samples appear evidently bigger than those from contemporaneous dogs (De Grossi Mazzorin and Tagliacozzo, 2000). For this reason, they were classified as wolves, as later confirmed also by the genetic analysis presented in this contribution.

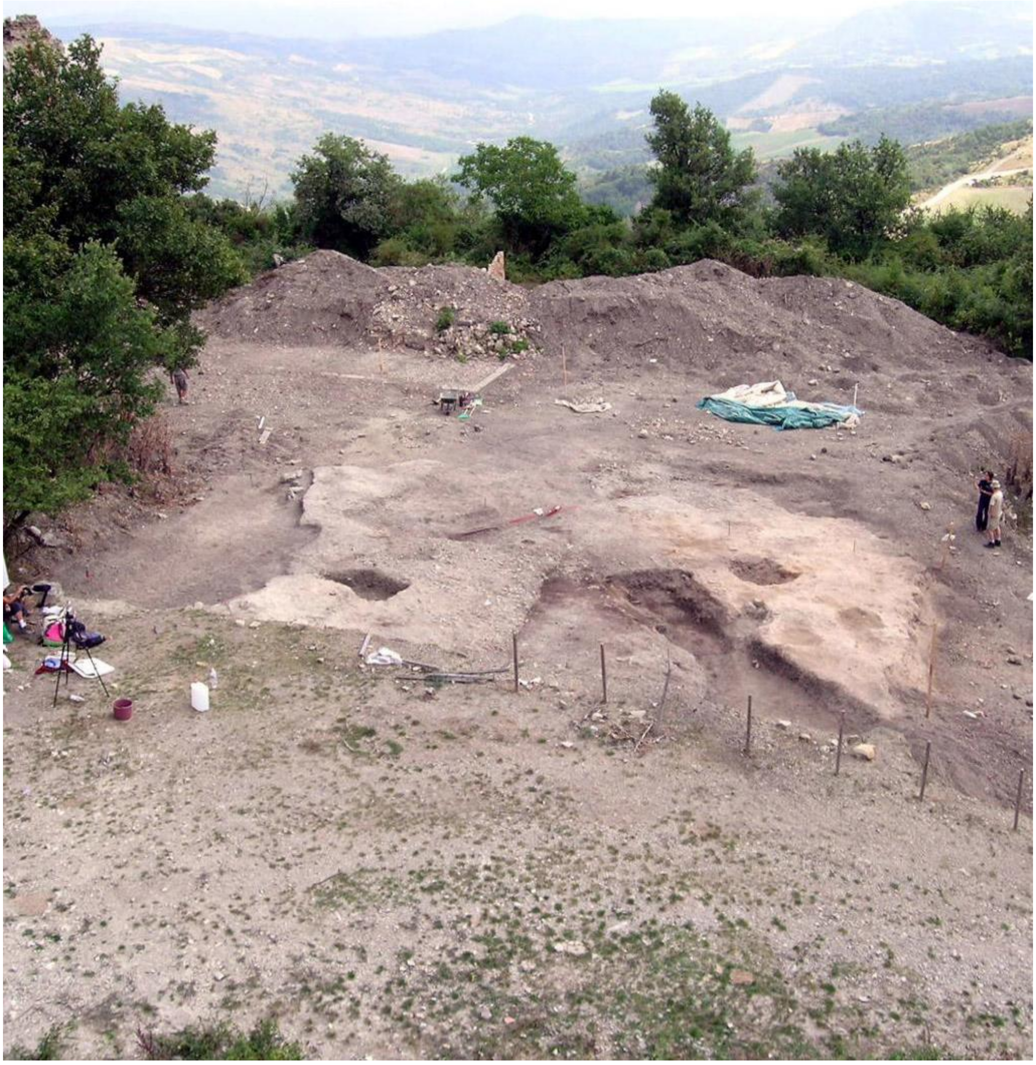

Figure S5. Monterenzio Vecchio archaeological site, where the samples OWW17 and OWW18 were excavated.
Photo credit: Vanessa Poli.

**3. Po River site**

The wolf fossil skull (OWW19) was discovered on the alluvial bar of the Po River right bank near Coltaro (PR) in Lombardy region (Northern Italy) (Fig. S2 and Fig. S6). A “bar” represents an elevated region of a meandering river, where the flow allows the deposit of sediments. The heavy particles of the sediment deposit more easily on the riverbed also in specific areas of the river where the flow is less intense. In this case, a skull or bone meeting these deposits get trapped and contribute to increasing the size/height of the bar. The ecosystem of a meandering river is highly variable, and the erosion process plays an important role in the releasing of material from the alluvial bars, thanks also to atmospheric events (Persico *et al.*, 2015).

The area, which is well-known for its numerous paleontological Quaternary record (Persico *et al.*, 2006), composes of a crescent-shaped meander bar (about 500 meters), located upstream the meander of Isola Maria Luigia, few kilometres from the city of Casalmaggiore (CR).

The fossil skull of this paper is a rare and well-preserved discovery in the Po Valley.

The clear brown beige colouring of the bones and the white orange hue of the teeth is typical of the evidence found in the alluvial sediments of the Po river, namely partially mineralized fossil bones containing pyrite, limonite, hematite, manganese, and manganocalcite (Persico *et al.*, 2012). The abundance of these minerals indicates a primary anoxic environment of fossilization and a subsequent one oxidising environment in the form of the river.

The fossil shows evidence of river transport (rafting) including post mortem fractures of the bones and the absence of canine and incisive teeth. The well-preserved state of the fossil reveals every diagnostic trait necessary for its specific classification.

The morphometric study about this sample was performed using manual measuring instruments such as a manual gauge, an anatomical compass with curved branches, a level, and a yardstick. Angular measurements were obtained using a manual goniometer and Corel Draw 16 application.

In the morphological description, we used the same biometric measurements and indexes adopted by Andersone and Ozolins (2000), Boudadi-Maligne and Escarguel (2014), compared with the database proposed by Andersone and Ozolins (2000) and Siracusa and Lo Valvo (2005). The diagnostic value of the orbital angle was compared with the same measurement of comparative *Canis lupus familiaris* skull and the values proposed by Janssens et al. (2016). The biological age of the specimen was also estimated by considering the tooth wear and the conditions of cranial suture welding (Gipson *et al.*, 2000).

A prominent sagittal crest protruding over the occipital condyles for 20.17 mm characterizes the wide and massive skull. The rostrum is long, and the zygomatic arcades are wide. In the frontal

view, the skull has a triangular shape and the orbital angle is approximately 40°; laterally it is possible to observe the absence of a frontal jump. The studied skull shows 12 superior teeth represented by I3, Pm 1,2,3,4; M 1,2 left and Pm 2,3,4; M1,2 right. The Total Lenght of skull is 228,57 mm (ToL) and the Height is 81,41 mm (SH). The width of the neuro-cranium at its widest point is 60,14 mm (GbW) while in the narrower one it measures 39,26 mm (LB). The 26 biometric measures adopted are the same used by Andersone and Ozolins (2000) and Boudadi-Maligne and Escarguel (2014).

The data set and the observed morphology result are compatible with the taxonomical attribution to *Canis lupus*.

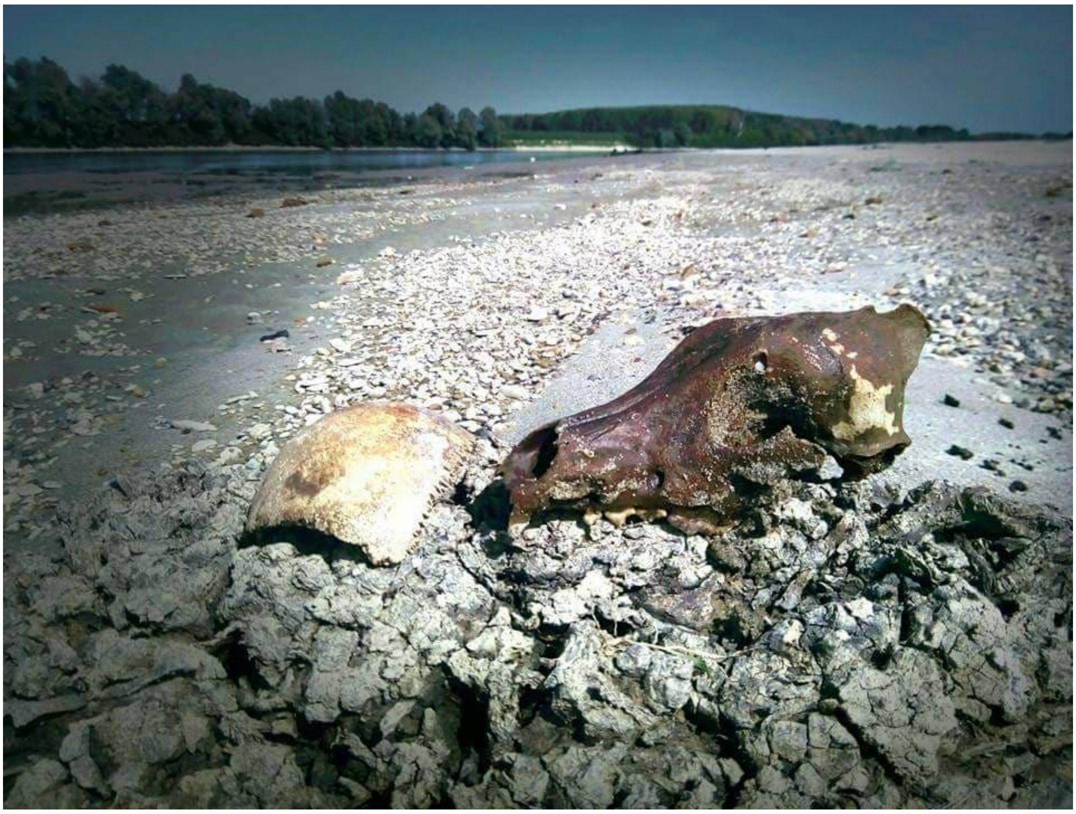

Figure S6. Area of the discovery of the sample OWW19 emerged from an alluvial bar of the Po River in province of Cremona, northern Italy.

Photo credit: Davide Persico.

***Radiocarbon Dating Analysis of the Po River sample***

The fossil was found in the typical allochthone position. For this reason, there are no stratigraphic information and the dating was possible only with a radiometric method. The dating of this sample was performed by Accelerator Mass Spectrometry (AMS) by using a 3MV Tandetron accelerator at

CEDAD-CEnter for Dating and Diagnostics (Lecce, Italy), to determine the absolute chronology by using the radiocarbon 14C method. This analysis provided a dating of ca. 983 - 793 BP (cal 2$\sigma$) (Table S1 and Fig. S7).

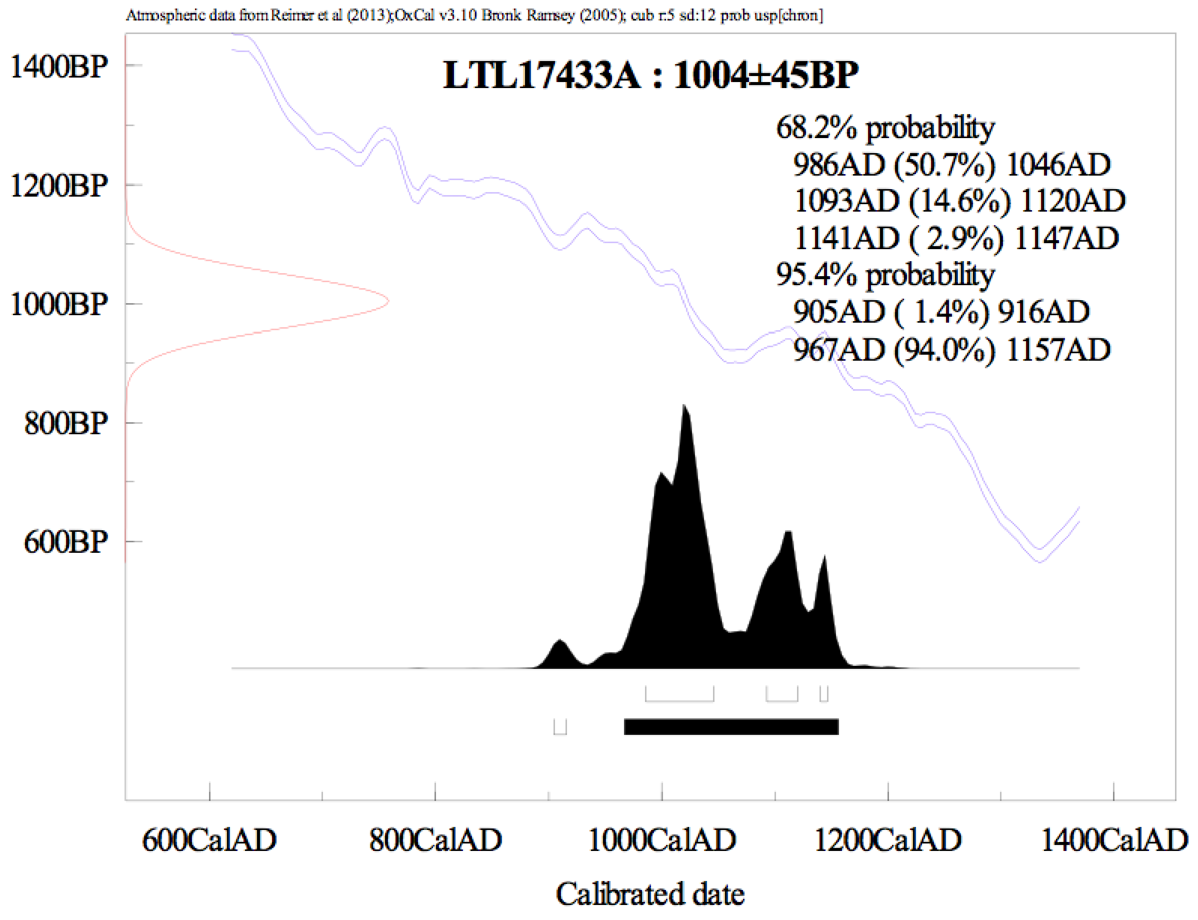


Figure S7. Calibration graph representing the radiocarbon age of the OWW19 sample. With a probability of 95.4% this sample was assigned to a chronological range of 1004±45 BP.

## Protocols of ancient DNA analysis

*DNA extraction*

The DNA was extracted twice for each sample using a protocol modified from literature (Dabney, Meyer and Paabo, 2013; Allentoft *et al.*, 2015). A minimum of 44 mg to 500 mg of powder from each sample was incubated overnight under constant rotation at 37° C in an extraction buffer (0.45M EDTA pH 8.0, 0.25mg/ml proteinase K) along with an extraction blank for each batch of samples. Then the supernatant, mixed with the PB buffer solution (Qiagen, GmbH, Hilden, Germany), was purified using the silica spin-columns and PE buffer (Qiagen, GmbH, Hilden, Germany). The final elution was performed in 50 µl of TET buffer.

*DNA amplification and sequencing*

Two independent PCRs were performed for each extraction, for each of the different set of primers. To detect contamination and decrease the risk of cross-contamination during PCR preparations, all the amplifications were performed for small groups of samples, along with the respective extraction blanks and PCR controls.

The amplification of the hypervariable region 1 (HVR1) of the mitochondrial DNA (mtDNA) was performed by using different sets of primers.

The amplification of the 99 bp fragment was performed using primers "HVR1-wolf-F'' (5'-ATA TTA TAT CCT TAC ATA GGA CAT-3') and ''HVR1-wolf-R'' (5'-ATT AAG CCC TTA TTG GAC T-3') (Stiller *et al.*, 2006). The amplification mix was prepared using: 2.5mM $MgCl_2$ (Thermo Fisher Scientific), 0.25mM dNTPs, 0.2μM of each primer, 1X PCR-Gold buffer (Thermo Fisher Scientific), 0.8mg/ml BSA, 2.5 units of AmpliTaq Gold (Thermo Fisher Scientific), ultrapure water and 4μl of DNA extract to give a total volume of 25μl/reaction. Amplifications were run in the post-PCR laboratory, with an initial denaturation step at 94 °C for 3 min, 60 cycles consisting of 30 s at 94 °C, 30 s at 50 °C, 30 s at 72 °C for 30 s, followed by a final extension at 72 °C for 15 min.

The amplification of the 361 bp stretch was obtained by means of three overlapping fragments with the primers:

1) MS_wolf1 (5'-TGCTCCACCATCAGCACCCA-3') - dogdl5 (5'-CATTAATGCACGACGTACATAGG-3') - 148bp (Leonard, Vilà; and Wayne, 2005);
2) MS_wolf2F (5'-TGAATCACCCCTACTGTGCT-3') - MS_wolf2R (5'-AGCCCTTATTGGACTAAGTGATATGC-3') - 205 bp (Ersmark *et al.*, 2016);
3) MS_wolf3F (5'-ACATAGGACATATTAACTCAATCTCAT-3') - MS_wolf3R (5'-TGGCCCTGAGGTAAGAACCAGA-3') - 211 bp (Ersmark *et al.*, 2016).

Excluding primers, the three overlapping fragments covered the following nucleotide positions of the Italian wolf mitochondrial genome (Genbank accession number KU644662):

1) 15431 – 15537
2) 15502 – 15660
3) 15631 – 15792

PCR mix was made using: 1X PCR-buffer (Qiagen), 1 mM MgCl2 (Qiagen), 0.2 mM dNTPs, 0.2 μM of each primer, 0.8 mg/ml BSA, 2.5 units of AmpliTaq Gold (Thermo Fisher Scientific), ultrapure water and 2μl of DNA extract, in a total volume of 25 μl. PCR cycles were set up with 10 min at 95 °C, then 55 cycles at 95 °C for 30 s, 30 s at 58–62 °C, and 30 s at 72 °C, followed by a

final extension step at 72 °C for 7 min.

Amplification products were checked using agarose gel electrophoresis and further purified using the MinElute PCR Purification™ kit (Qiagen GmbH, Hilden, Germany), following manufacturer instructions. The purified amplicons of the first independent PCR reactions were sequenced at the Conservation Genetics Area of Istituto Superiore per la Protezione e la Ricerca Ambientale (ISPRA) (Ozzano dell'Emilia, Bologna, Italy) and the products of the second independent amplifications were sequenced at the laboratories of Pharmacogenetics and Pharmacogenomics of the Department of Pharmacy and Biotechnology (University of Bologna). In both laboratories, sequencing reactions were conducted in both directions using the forward and the reverse primers and the BigDye Terminator kit ver.1.1 (Applied Biosystems Inc.). All the PCR products were purified with the DyeEx 96 Kit (Qiagen) or by ethanol and sodium acetate DNA precipitation and finally sequenced using an Applied Biosystems 3130XL Genetic Analyzer automatic sequencer.

**References**

Allentoft, M. E. et al. (2015) 'Population genomics of Bronze Age Eurasia', Nature, 522, pp. 167–172. doi: 10.1038/nature14507.

Andersone Z., Ozolins J. 2000. Craniometrical characteristics and dental anomalies in wolves Canis lupus from Latvia. *Acta Theriologica* 45:549–558.

Boudadi-Maligne M., Escarguel G. 2014. A biometric re-evaluation of recent claims for Early Upper Palaeolithic wolf domestication in Eurasia. *Journal of Archaeological Science* 45:80–89.

Dabney, J., Meyer, M. and Paabo, S. (2013) 'Ancient DNA Damage', Cold Spring Harbor Perspectives in Biology, 5(7), pp. a012567–a012567. doi: 10.1101/cshperspect.a012567.

De Grossi Mazzorin, J. , Tagliacozzo, A. (2000) Morphological and osteological changes in the dog from the Neolithic to the Roman Period in Italy, in Crockford S. J. (ed.), *Dogs Through Time: An Archaeological Perspective (International Congress of the International Council for Archaeozoology (ICAZ '98), Victoria, BC, Canada 23-29 Agosto 1998)* (BAR International Series 889). Archaeopress, Oxford. pp. 141-161.

Driesch, von Den A. (1976) *A guide to the measurement of animal bones from archaeological sites: as developed by the Institut für Palaeoanatomie, Domestikationsforschung und Geschichte der Tiermedizin of the University of Munich* (Volume 1 of Peabody Museum Monographs). Peabody Museum of Archaeology and Ethnology, Cambridge, MA.

Ersmark, E. et al. (2016) 'From the Past to the Present: Wolf Phylogeography and Demographic History Based on the Mitochondrial Control Region', Frontiers in Ecology and Evolution,

4(December). doi: 10.3389/fevo.2016.00134.

Gipson PS., Ballard WB., Nowak RM., Mech LD. 2000. Accuracy and precision of estimating age of gray wolves by tooth wear. Journal of Wildlife Management 64:752–758.

Guerra, L. et al. (2010) 'L'abitato d'altura dell'Età del Bronzo di Monterenzio Vecchio (Bologna)', in XLV Riunione Scientifica dell'Istituto Italiano di Preistoria e Protostoria, Preistoria e Protostoria dell'Emilia Romagna. Modena.

Janssens L., Miller R., Van Dongen S. 2016. The morphology of the mandibular coronoid process does not indicate that Canis lupus chanco is the progenitor to dogs. Zoomorphology 135:269–277. DOI: 10.1007/s00435-015-0298-z.

Leonard, J. A., Vilà, C. and Wayne, R. K. (2005) 'Legacy Lost: Genetic variability and population size of extirpated US gray wolves.', Molecular Ecology, (126), pp. 198–206.

Maini, E. (2012) Lo sviluppo dell'allevamento in Emilia-Romagna: Aspetti economici e implicazioni sociali nella gestione della risorsa animale durante l'età del Bronzo. Università di Bologna. Available at: http://amsdottorato.unibo.it/5060/.

Nenzioni, G., Marchesini, M. and Marvelli, S. (2018) 'Fenomeni carsici e primo popolamento nel territorio bolognese orientale: paleoambienti e litocomplessi', in P. Boccuccia, R. Gabusi, C. Guarnieri, M. Miari (a cura di), "…. nel sotterraneo Mondo". La frequentazione delle grotte in Emilia-Romagna tra archeologia, storia e speleologia, Atti del convegno (Brisighella 2017), FSRE Editore, pp. 21-31.

Paronuzzi, P., Berto, C., Ghezzo, E., Thun Hohenstein, U., Massarenti, A., Reggiani, P. (2018) 'Nota preliminare sulla sequenza UMG di ex Cava a Filo (Croara, BO): gli aspetti stratigrafico-sedimentari, paleontologici e antropici alla luce delle ultime indagini (2006-2016)', Memorie dell'Istituto Italiano di Speleologia, 2 (32): 131-144.

Pasini, G. (1970) 'Contributo alla conoscenza del tardo wurmiano e del postwurmiano nei dintorni di Bologna', Giornale di Geologia, 2, pp. 687–700.

Pasini, G. (1969) 'Fauna a mammiferi del Pleistocene Superiore in un paleoinghiottitoio carsico presso Monte Croara (Bologna)', Le Grotte d'Italia, 4, pp. 1–46.

Persico, D. et al. (2006) I Fossili del Po: catalogo dei mammiferi fossili delle alluvioni quaternarie del Museo Naturalistico Paleontologico di San Daniele Po (CR). Persico Dosimo (CR): Edizioni DelMiglio.

Persico, D. et al., (2015) The skull of Stephanorhinus kirchbergensis (Jäger, 1839) (Mammalia, Rhinocerotidae) from Spinadesco (Cremona, Lombardia, Northern Italy): morphological analyses and taxonomical remarks – an opportunity for revising the three other skulls from the Po Valley. Quaternary Science Reviews, 109, pp.28-37.

Siracusa AM., Lo Valvo M. 2005. Confronti craniometrici tra lupi (Canis lupus) dell'Italia continentale e della Spagna: primi dati. Hystrix-the Italian Journal of Mammalogy 15.

Stiller, M. et al. (2006) 'Patterns of nucleotide misincorporations during enzymatic amplification and direct large scale sequencing of ancient DNA', Proceedings of the National Academy of Sciences, 103(37), pp. 13578–13584. doi: 10.1073/pnas.0605327103.
